# Fgfr3–Wnt signaling crosstalk is involved in maintaining cranial suture integrity

**DOI:** 10.1038/s41413-026-00558-w

**Published:** 2026-07-30

**Authors:** Rachel Pereur, Yvan Marc, Yuliya Lim, Alain Schmitt, Marilyne Malbouyres, Anatole Chessel, Florence Ruggiero, Marie-Claire Schanne-Klein, Laurence Legeai-Mallet, Emilie Dambroise

**Affiliations:** 1 Laboratory of Genetics of Developmental Disorders, INSERM, UMR1163, Institut Imagine, Université Paris Cité, Paris, France; 2 Laboratory for Optics and Biosciences, Ecole Polytechnique, CNRS, INSERM, Institut Polytechnique de Paris, Palaiseau, France; 3 Université Paris Cité, CNRS, Inserm, Institut Cochin, Paris, France; 4 Institut de Génomique Fonctionnelle de Lyon, Université de Lyon, ENS de Lyon, CNRS UMR5242, Université Claude Bernard Lyon 1, Lyon, France

**Keywords:** Bone, Diseases

## Abstract

Cranial suture formation is a dynamic process that requires precise cellular and molecular coordination to regulate bone growth and maintain suture homeostasis. The Fibroblast Growth Factor Receptor 3 (FGFR3) signaling pathway is among the major pathways disrupted in craniosynostosis; however, its precise role during cranial suture formation is still unknown. Using a relevant *fgfr3* LoF zebrafish model exhibiting abnormal suture morphology, we demonstrated for the first time that Fgfr3 plays a pleiotropic role in both the formation and maintenance of cranial sutures. Transmission electron microscopy and second harmonic generation imaging revealed that Fgfr3 is essential for the proper organization of the collagen network within the suture. Using specific transgenic reporter lines, we showed that Fgfr3 is crucial for regulating osteogenesis in this region. Specifically, Fgfr3 limits the number of osteoprogenitors at the osteogenic front and promotes osteoblast maturation at the suture edge. RNAscope analyses further revealed that loss of Fgfr3 led to significant upregulation of *fgf18* expression. Finally, our findings show that loss of Fgfr3 results in the activation of the canonical Wnt pathway, and possibly the BMP pathway, within cranial sutures. Pharmacological inhibition of canonical Wnt signaling during suture development using the β-catenin inhibitor XAV939 restored *fgf18* expression, partially normalized levels of the BMP antagonist *grem1*, reduced SMAD1/5 phosphorylation, and produced a significant improvement in cranial suture morphology. In conclusion, these findings position Fgfr3 as a central regulator of cranial suture formation and homeostasis, acting through intricate cross-talk between the FGF, canonical Wnt, and possibly BMP signaling pathways. These data offer new insights into the biology of cranial suture and FGFR3-related craniosynostoses.

## Introduction

Cranial sutures are essential structures in skull development. Acting as fibrous joints between rigid calvarial bones, they provide plasticity for skull expansion during early brain growth and contribute to skull shape, symmetry, and absorption of mechanical forces through adulthood.^1^ In humans, mice, and zebrafish, five primary sutures extend from anterior to posterior, delineating the boundaries between cranial bones: the metopic suture, the two coronal sutures, the sagittal suture, and the lambdoid suture.^2^ Their formation is a dynamic process requiring precise cellular and molecular coordination to regulate bone growth and suture homeostasis. At the edges of the approaching bones, an osteogenic front (OF) composed of osteoprogenitor cells is established. These proliferating cells sequentially differentiate into immature and mature osteoblasts forming new bone matrix that is initially restricted to growing bone tips and then at the edges of the suture.^3–5^ To support this process, a heterogeneous population of suture mesenchymal stem cells (SuSCs), expressing various markers such as Gli1, Axin2, Prrx1, Ctsk, Bmpr1a, Ddr2, and Grem1a, resides between the OFs, preserving suture patency in a fibrous, unossified state while also contributing to the recruitment of osteoprogenitors at the ossification front. Each subpopulation presents their own properties and participates more or less in the osteogenesis within the OF.^5–12^ All these cells are surrounded by an extracellular matrix (ECM) rich in collagens, which provides structural support and participates in the transmission of biomechanical signals, as sutures are subjected to significant tensile and compressive forces, both essential for the regulation of osteogenesis.^13–18^

Several signaling pathways and genes involved in suture homeostasis have been implicated in genetic diseases characterized by pathological suture expansion or premature fusion, known as craniosynostosis. This includes Fibroblast Growth Factor Receptor 3 (FGFR3), where both gain-of-function (GoF) and loss-of-function (LoF) mutations lead to distinct cranial suture defects. GoF mutations are associated with syndromic craniosynostosis, as observed in Muenke syndrome, one of the most frequent forms of craniosynostosis, and in Crouzon syndrome with acanthosis nigricans.^19,20^ In contrast, LoF mutations result in Camptodactyly-Tall Stature-Scoliosis-Hearing Loss (CATSHL) syndrome, which leads to the formation of ectopic bones along the sutures, known as Wormian bones.^21^ However, despite these findings, *Fgfr3* mouse models with GoF or LoF mutations have failed to fully recapitulate these cranial phenotypes, posing a significant challenge in deciphering the precise role of FGFR3 in cranial suture formation and maintenance.^22–24^ Zebrafish, widely used to study craniofacial development in normal and pathological contexts (including craniosynostosis), has emerged as a relevant model for investigating Fgf signaling and Fgfr3 function during cranial vault formation.^5,11,25,26^ Interestingly, similar to CATSHL syndrome, *fgfr3* LoF zebrafish exhibit microcephaly and Wormian bones.^27,28^ Our previous data on a *fgfr3* LoF zebrafish model (*fgfr3*^*lof/lof*^*)* demonstrated that *fgfr3* is expressed in late-stage osteoprogenitors and both immature and mature osteoblasts, and regulates positively both the expansion and maturation of immature osteoblasts during cranial vault bone development.^28^ Interestingly, at adult stage *fgfr3*^*lof/lof*^ zebrafish exhibit a dramatic impairment of metopic and sagittal sutures formation, with bones that fail to overlap and remain separated by a thick layer of fibrous tissue. This phenotype, which contrasts with craniosynostosis, makes *fgfr3*^*lof/lof*^ fish an ideal model for studying the involvement of Fgfr3 in cranial suture homeostasis.

Here, to achieve this goal, we analyzed the key factors involved in the formation and maintenance of cranial sutures in *fgfr3*^*lof/lof*^ zebrafish, from suture initiation at 12 standard length (SL) to adulthood (20 and 26 SL). We showed that Fgfr3 loss completely disrupts suture formation, affecting not only the structural integrity of the sutures but also the organization of the collagen network within the sutures and at the bone ends, as shown by transmission electron microscopy (TEM) and second harmonic generation (SHG) imaging. To further dissect the impact on osteogenesis, we used transgenic lines marking osteoprogenitors [*Tg(runx2:*GFP*)*], immature [(*Tg(sp7:*mCherry*)*] and mature [*Tg(bglap:*GFP*)*] osteoblasts, and showed that Fgfr3 deficiency affects the entire membranous ossification process involved in suture formation and maintenance. In parallel, using RNAscope in situ hybridization to detect SuSC markers and the Wnt reporter line *Tg(7xTCF-Xla.Siam:GFP)*^*ia4*^, we demonstrated that loss of Fgfr3 leads to aberrant activation of the canonical Wnt signaling pathway, and potentially the BMP pathway, within cranial sutures. Finally, to test whether canonical Wnt signaling mediates the suture phenotype observed in *fgfr3*^*lof/lof*^ fish, we treated mutants with the β-catenin inhibitor XAV939 during cranial vault development and observed a partial rescue of the suture defects. Our study provides new insights of the role of Fgfr3 into suture homeostasis.

## Results

### Fgfr3 is required for proper cranial suture formation

**Fig. 1:**
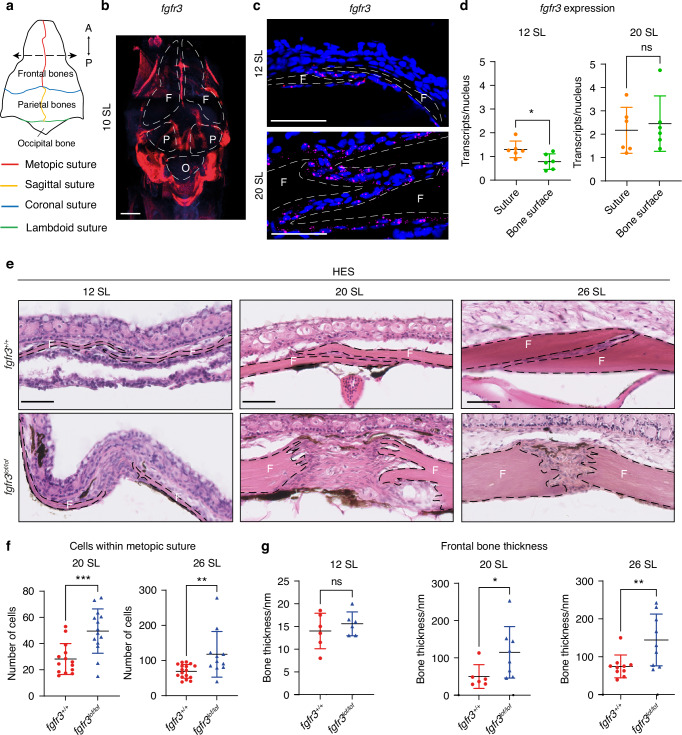
Fgfr3 is required for cranial vault suture formation. **a** Schematic representation of the zebrafish cranial vault. The dotted arrow corresponds to the plane of sectioning. **b** *fgfr3* expression (red) on whole mount *fgfr3*^*+/+*^ cranial vault at 10 SL labeled by RNAscope in situ hybridization showed the high expression of *fgfr3* at the periphery of cranial bones and in the forming sutures. F = Frontal bone, P = Parietal bone, O = Occipital bone. Scale bar = 300 µm. **c** *fgfr3* expression (red) on coronal sections of *fgfr3*^*+/+*^ metopic suture at 12 and 20 SL labeled by RNAscope in situ hybridization. At both stages, *fgfr3* was expressed in cells along the bone surface and within the suture. Nuclei were counterstained with DAPI (blue). Scale bar = 50 µm. **d** RNAscope, quantitative analyses of the number of transcript (dot) relative to the number of nuclei (DAPI) highlighted that at 12 SL *fgfr3* was more highly expressed inside the suture compared to the bone surface. In contrast, at 20 SL, no difference of *fgfr3* expression was observed based on localization (12 and 20 SL: *fgfr3*^*+/+*^ n = 6; *fgfr3*^*lof/lof*^ *n* = 6). **e** HE stained coronal sections of the zebrafish head at the metopic suture level at 12, 20, and 26 SL demonstrated that the absence of Fgfr3 induced a delay of frontal bone growth, and prevented frontal bones overlap at 20 and 26 SL. The dotted lines represent the bone boundaries. Scale bar = 50 µm. **f** Quantification of the number of cells inside the suture at 20 SL (*fgfr3*^*+/+*^ *n* = 14; *fgfr3*^*lof/lof*^ *n* = 14) and 26 SL (*fgfr3*^*+/+*^ *n* = 17; *fgfr3*^*lof/lof*^ *n* = 11) showed that the *fgfr3*^*lof/lof*^ fish present a greater number of cells within the suture at adulthood. **g** Measurement of frontal bone thickness close to the metopic suture at 12 SL (*fgfr3*^*+/+*^ *n* = 6; *fgfr3*^*lof/lof*^ *n* = 6*)*, 20 SL (*fgfr3*^*+/+*^ *n* = 6; *fgfr3*^*lof/lof*^ *n* = 8) and 26 SL (*fgfr3*^*+/+*^ *n* = 10; *fgfr3*^*lof/lof*^ *n* = 9*)* showed that the *fgfr3*^*lof/lof*^ bones are thicker than *fgfr3*^*+/+*^ bones at adulthood. F: frontal bone. Data are presented as mean ± SD. The *P*-values were determined by Student’s *t* tests: ns: not significant; *P < 0.05; **P < 0.01; ***P < 0.001

In adult wild-type zebrafish, metopic, sagittal and coronal sutures (Fig. 1a) present the same structure as human coronal sutures, with overlapping bones, osteogenic cells at their edge and a central layer of SuSCs within collagen-rich ECM. We previously reported that *fgfr3*^*lof/lof*^ metopic and sagittal sutures displayed a failure of bone overlap, with the bones being separated by a thick fibrous tissue at 20 SL.^28^ As the metopic suture is among the first to develop in zebrafish, we focused on the role of Fgfr3 during formation of this suture. We used RNAscope in situ hybridization to map *fgfr3* expression during metopic suture formation in wild-type fish at three stages: 10 SL (frontal bones approaching), 12 SL (suture formation), and 20 SL (suture maintenance, adult stage) (Fig. 1b, c). At 10 SL, *fgfr3* was expressed at the OF of the developing cranial bones, and on the forming metopic suture (Fig. 1b). At later stages, transversal sections showed *fgfr3* expression within the suture and in osteogenic cells above and below the frontal bones, a region we will refer to as the bone surface (Fig. 1c). At 12 SL, we noted that *fgfr3* expression was higher within the suture compared to the bone surface, whereas by 20 SL, expression levels were comparable in both regions (Fig. 1d).

To investigate metopic suture progression in *fgfr3*^*lof/lof*^ fish over time, we examined it at 12, 20 and 26 SL (Fig. 1e). While *fgfr3*^*+/+*^ fish displayed a well-formed suture at 12 SL, *fgfr3*^*lof/lof*^ frontal bones remained separated in agreement with our previous data.^28^ By 20 and 26 SL, although the bones sometimes came closer together, they still did not overlap in the mutants (Fig. 1e). Interestingly lack of bone overlap in *fgfr3*^*lof/lof*^ was linked to increased cell number in the metopic suture and thicker bones at 20 and 26 SL compared to controls (Fig. 1f, g). Moreover, sagittal sections at 20 SL revealed that coronal sutures were also affected, indicating that Fgfr3 loss impacts all cranial vault sutures similarly (Fig. S1a). Bone thickening was observed in coronal bones as well at 20 and 26 SL (Fig. S1b).

These findings demonstrate that the absence of *fgfr3* disrupts proper cranial suture causing bones to abut rather than overlap throughout the lifespan of zebrafish and leads to bone expansion, predominantly in thickness rather than length.

### Fgfr3 loss leads to a progressive disruption of collagen fibrils within the suture

**Fig. 2:**
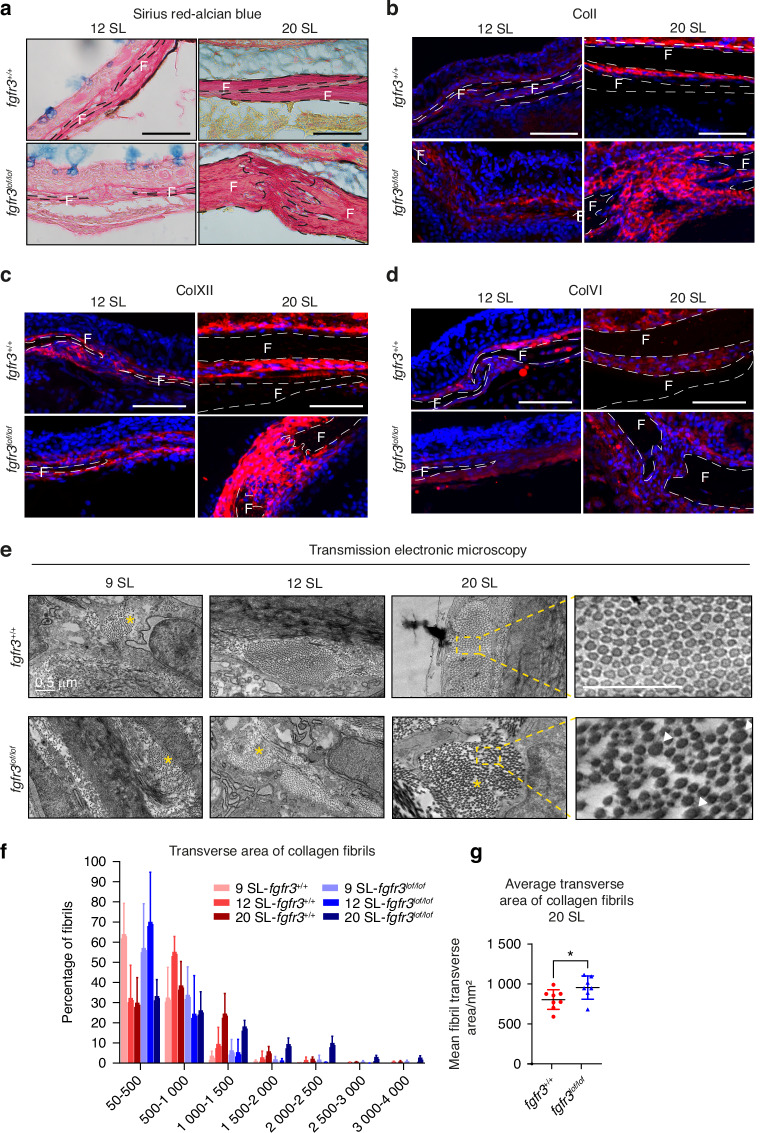
Fgfr3 deficiency leads to disruption of fibrillogenesis within the suture at adulthood. **a** Sirius Red–Alcian Blue stained coronal sections of the zebrafish head at the metopic suture level at 12 and 20 SL demonstrated that the absence of Fgfr3 induced an accumulation and a disorganization of the suture ECM at 20 SL. The dotted lines represent the bone boundaries. Scale bar = 50 µm. Immunofluorescence against ColI (**b**), ColXII (**c**) and ColVI (**d**) on metopic sutures of *fgfr3*^*+/+ or lof/lof*^ fish at 12 and 20 SL showed a higher amount of ColI and ColXII within the suture of the *fgfr3*^*lof/lof*^ fish. Nuclei were counterstained with DAPI (blue) and the dotted lines represent the bone boundaries. Scale bar = 50 µm. (12 SL: ColI: *fgfr3*^*+/+*^ *n* = 3; *fgfr3*^*lof/lof*^ *n* = 5; ColVI: *fgfr3*^*+/+*^ *n* = 7; *fgfr3*^*lof/lof*^ *n* = 5; ColXII: *fgfr3*^*+/+*^ *n* = 3; *fgfr3*^*lof/lof*^ *n* = 3) (20 SL: ColI: *fgfr3*^*+/+*^ *n* = 3; *fgfr3*^*lof/lof*^ *n* = 3; ColXI: *fgfr3*^*+/+*^ *n* = 5; *fgfr3*^*lof/lof*^ *n* = 5; ColXII: *fgfr3*^*+/+*^ *n* = 3; *fgfr3*^*lof/lof*^ *n* = 3). **e** Coronal sections of 9, 12, and 20 SL fish imaged by transmission electron microscopy. Disorganized fibril clusters and enlarged abnormal fibrils are indicated by yellow asterisks and white arrowheads, respectively. Scale bar = 0.5 µm. **f** Measurement of fibril transverse area distribution within the suture at 9, 12, and 20 SL revealed a more heterogeneous fibril size in *fgfr3*^*lof/lof*^ compared to controls. **g** Comparison of the mean fibril transverse area in the metopic suture of *fgfr3*^*+/+ and lof/lof*^ fish revealed an increase in fibril size in the mutants at 20 SL (9 SL: (*fgfr3*^*+/+*^ *n* = 3; *fgfr3*^*lof/lof*^ *n* = 2) (12 SL: (*fgfr3*^*+/+*^ *n* = 3; *fgfr3*^*lof/lof*^ *n* = 3) (20 SL: (*fgfr3*^*+/+*^ *n* = 2; *fgfr3*^*lof/lof*^ *n* = 2). F: Frontal bone. Data are presented as mean ± SD. The *P*-value was determined by a Student’s *t* test: *P < 0.05

Cells within cranial vault sutures of *fgfr3*^*lof/lof*^ fish are surrounded by a dense ECM rich in collagens, as shown by Sirius red-Alcian blue staining (Fig. 2a). In our previous study, we found that the absence of Fgfr3 during cranial vault development leads to the upregulation of the expression of several ECM-regulating genes, including *col6a1*, *col12a1a*, and *col12a1b*.^28^ To assess this persists in sutures, we performed immunofluorescence for ColVI, ColXII, and ColI which is also known to be expressed in cranial sutures. We observed that ColI and ColXII were expressed within the cranial sutures of both *fgfr3*^*+/+*^ and *fgfr3*^*lof/lof*^ fish at 12 SL, and that the expression levels of both proteins are increased only in older *fgfr3*^*lof/lof*^ fish (20 SL) (Fig. 2b, c). In contrast, ColVI was expressed to a lesser extent at both stages and genotypes (Fig. 2d). As ColXII is known to interact with ColI fibrils and to regulate collagen fibril spacing and assembly during fibrillogenesis, we analyzed collagen fibrils in the metopic suture by TEM at 9, 12, and 20 SL (Fig. 2e).^29^ At 9 SL, sutures were unformed and fibrils disorganized in both genotypes (area marked with a yellow asterisk). By 12 SL, controls showed formed sutures with organized fibril clusters, while mutants had separated frontal bones and disorganized fibrils, similar to 9 SL. At 20 SL, *fgfr3*^*lof/lof*^ fibrils clustered like controls, but were less organized and more heterogeneous in size, confirmed by fibril transverse area measurements (Fig. 2e, f). Controls showed progressive fibril size increase, mostly under 1 000 nm² at 20 SL, whereas mutants had unchanged size at 9 and 12 SL, consistent with delayed bone closure, and significantly larger and heterogeneous fibrils at 20 SL (marked by white arrowheads), with some exceeding 2 500 nm². This increase was further confirmed by the larger average collagen fibril area in *fgfr3*^*lof/lof*^ at 20 SL compared to controls, showing an impairment of fibrillogenesis in adult sutures (Fig. 2g).

**Fig. 3:**
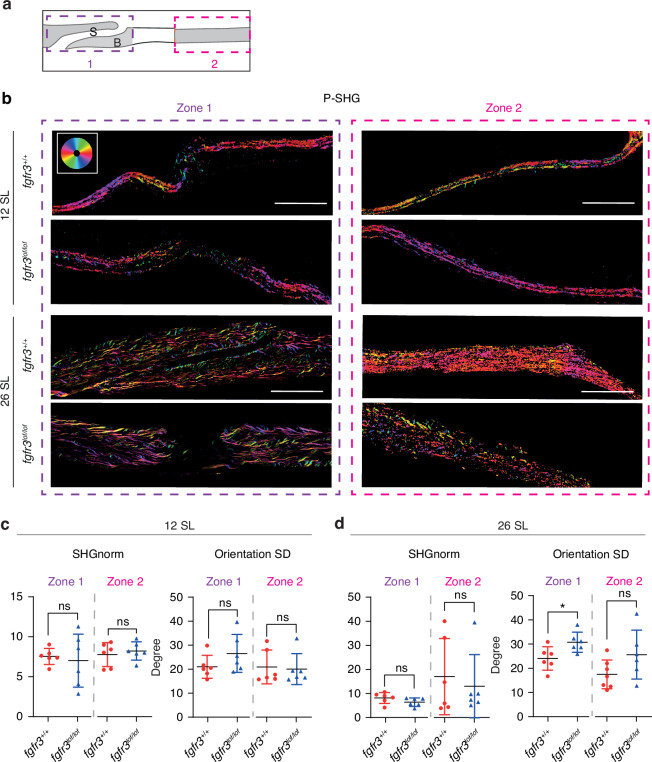
Loss of Fgfr3 affects the organization of bone collagen fibers at the bone ends over time. **a** Diagrams representing the different areas analyzed by SHG microscopy. Zone 1 corresponds to the bone surrounding the suture called the bones ends, while zone 2 refers to bone located farther away. **b** Coronal sections of metopic suture at 12 and 26 SL imaged by SHG microscopy at 12 and 26 SL. Scale bar = 50 µm. The color codes for the collagen orientation as shown by the color wheel in the inset. Quantification of the SHG intensity normalized to the excitation power squared (SHG_norm_) and of the circular standard deviation of the collagen orientation distribution (orientation SD) in zone 1 and 2 at 12 SL (**c**) and 26 SL (**d**). In zone 1, the circular standard deviation of the collagen orientation distribution is significantly higher in *fgfr3*^*lof/lof*^ at 26 SL, suggesting a disorganization of the collagen fibers at the ends of the frontal bones (12 SL: *fgfr3*^*+/+*^ *n* = 6; *fgfr3*^*lof/lof*^ *n* = 6) (26 SL: *fgfr3*^*+/+*^ *n* = 6; *fgfr3*^*lof/lof*^ *n* = 6). Data are presented as mean ± SD. The *P*-values were determined by Student’s *t* tests: ns: not significant; *P < 0.05

Noting defects in collagen fibrils at the suture, and bone thickening near sutures, we examined whether collagen fibers in the frontal bones were also affected over time by Fgfr3 loss. We performed polarization-resolved SHG microscopy at 12 and 26 SL to study collagen fibers structure and organization, assessing the signal intensity and the circular standard deviation of the collagen orientation distribution in a region close to the suture (zone 1) and another farther away (zone 2) (Fig. 3a). The circular standard deviation of the collagen orientation distribution measures the degree of variability in the orientation of collagen fibers, providing insight into their alignment and organization at the scale of the image. By 12 SL, no defects in either the structure or the organization were observed in collagen fibers in both regions (Fig. 3b, c). In contrast, by 26 SL, although no defect in the collagen structure was noted, a significant disorganization of collagen fibers was observed only in the region adjacent to the suture (zone1) (Fig. 3b, d). The absence of structural defects and the progressive, localized disorganization of bone collagen fibers at the bone ends suggest that this defect is not directly due to the absence of Fgfr3 but may instead be secondary to changes in suture shape.

### The absence of Fgfr3 results in the excessive expansion of osteoprogenitors and inhibits the maturation of osteoblasts at the suture edge

**Fig. 4:**
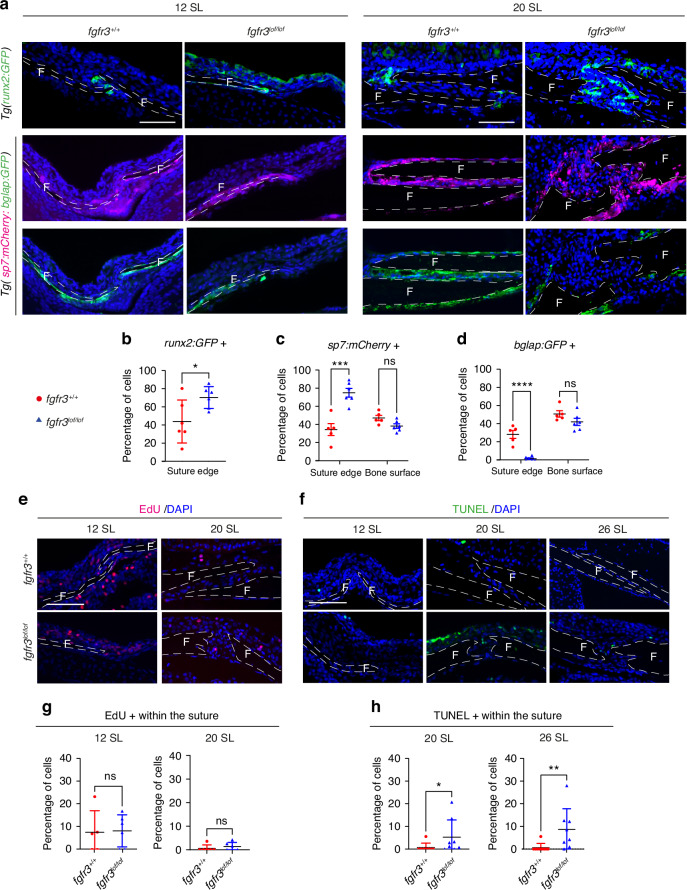
Fgfr3 prevents osteoprogenitor expansion, and activates osteoblast maturation within the metopic suture. **a** Immunofluorescence against GFP (green) and mCherry (red) was performed on metopic sutures of *Tg(runx2:GFP*; *fgfr3*^*+/+ or lof/lof*^) or *Tg(sp7:mCherry; bglap:GFP*; *fgfr3*^*+/+ or lof/lof*^) fish at 12 and 20 SL. Scale bar = 50 µm. **b** Quantification at 20 SL of the percentage of osteoprogenitors along the suture revealed a significantly higher number of osteoprogenitors in the *fgfr3*^*lof/lof*^ suture compared to the control (*fgfr3*^*+/+*^ *n* = 6; *fgfr3*^*lof/lof*^ *n* = 6). The *P*-value was determined by a two-way ANOVA. **c**, **d** Quantification at 20 SL of the percentage of *sp7*:mCherry positive cells and *bglap*:GFP positive cells along the suture and the bone surface revealed a greater number of immature osteoblasts and a drastically lower number of mature osteoblasts at the edge of the *fgfr3*^*lof/lof*^ metopic suture compared to controls. No significant difference was observed along the bone surface (*fgfr3*^*+/+*^ *n* = 5; *fgfr3*^*lof/lof*^ *n* = 6). The *P*-values were determined by a two-way ANOVA. **e** EdU assays performed on *fgfr3*^*+/+ or lof/lof*^ metopic suture at 12 and 20 SL. **f** TUNEL assays labeling apoptotic cells performed on *fgfr3*^*+/+*^ and *fgfr3*^*lof/lof*^ metopic suture at 12, 20, and 26 SL fish. Scale bar = 50 µm. **g** Quantification of the percentage of EdU positive cells revealed no significant defect of proliferation at 12 and 20 SL on *fgfr3*^*lof/lof*^ sutures (12 SL: *fgfr3*^*+/+*^ *n* = 5; *fgfr3*^*lof/lof*^ *n* = 6; 20 SL: *fgfr3*^*+/+*^ *n* = 6; *fgfr3*^*lof/lof*^ *n* = 6). *P*-values were determined by Student’s *t* tests. **h** Quantitative analysis of apoptotic cell percentages, relative to the number of nuclei stained with DAPI, revealed a significantly higher incidence of apoptosis in the *fgfr3*^*lof/lof*^ suture compared to *fgfr3*^*+/+*^ at 20 and 26 SL (20 SL: *fgfr3*^*+/+*^ *n* = 8; *fgfr3*^*lof/lof*^ *n* = 8; 26 SL: *fgfr3*^*+/+*^ *n* = 9; *fgfr3*^*lof/lof*^ *n* = 8). *P*-values were determined by Student’s *t* tests. Nuclei were counterstained with DAPI (blue) and the dotted lines represent the bones boundary. F: Frontal bone. Data are presented as mean ± SD. ns: not significant; *P < 0.05; **P < 0.01; ***P < 0.001; ****P < 0.000 1

To rule out excessive bone resorption as a cause for suture shape change, we first assessed osteoclast activity using tartrate-resistant acid phosphatase (TRAP) staining at 12, 20, and 26 SL. No TRAP positive cells were observed in the suture of either *fgfr3*^*+/+*^ or *fgfr3*^*lof/lof*^ fish at the stages tested (Fig. S2), confirming previous findings that osteoclasts do not contribute to suture homeostasis in zebrafish and excluding increased osteoclast activity as the cause of observed suture abnormalities.^30^ We then further explored osteogenesis using bone specific transgenic lines at the metopic suture from 12 SL to adulthood in *Tg(runx2:*GFP*; fgfr3*^*lof/lof or +/+*^*)* and *Tg(sp7:*mCherry*; bglap:*GFP*; fgfr3*^*lof/lof or +/+*^*)* lines (Fig. 4). At 12 SL, *runx2*:GFP positive osteoprogenitors in *fgfr3*^*+/+*^ fish were restricted to the OF at the tip of the bones, whereas in *fgfr3*^*lof/lof*^, they extended both to the bone tips and along the bone surface (Fig. 4a). By 20 SL, *runx2*:GFP positive cells remained at the bone tips, while in *fgfr3*^*lof/lof*^ fish they spread along the entire suture edge and were no longer detect on the bone surface (Fig. 4a). This redistribution was confirmed by a higher percentage of *runx2*:GFP positive cells at the suture edge in mutants (Fig. 4b). As we observed in controls that cells at the OF appear more circular than those at the suture edges and bone surface, we quantified nuclear circularity on these three sites (Fig. S3a–d). In control, as expected OF cell nuclei were significantly more circular than those at the suture edge and bone surface. However, in *fgfr3*^*lof/lof*^ fish, although bone surface nuclei were more elongated than control OF nuclei, nuclei present at the suture edge exhibited a circularity similar to control OF nuclei (Fig. S3b, d). These findings were further supported by TEM (Fig. S3c), indicating that cells at the suture edge in fgfr3^*lof/lof*^ fish exhibit features resembling those of OF cells in fgfr3^*+/+*^ fish, specifically a rounded nucleus and *runx2:*GFP positive.

Next, we studied the impact of the absence of Fgfr3 on immature (*sp7-positive*) and mature (*bglap-positive*) osteoblasts. In both *fgfr3*^*+/+*^ and *fgfr3*^*lof/lof*^ fish, immature osteoblasts were present throughout the bone surface and along the suture at all stages (Fig. 4a (12 and 20 SL) and Fig. S4a (26 SL)). The quantification of immature osteoblasts at 20 and 26 SL showed significantly more immature osteoblasts at the edge of the metopic suture in the *fgfr3*^*lof/lof*^ model compared to controls, with no difference along the bone surface (Figs. 4c, S4b). Regarding mature osteoblasts, in *fgfr3*^*+/+*^ fish we observed their presence throughout the bone surface and along the suture at all stages (Figs. 4d, S4c). Interestingly, in *fgfr3*^*lof/lof*^, while mature osteoblasts were present throughout the bone surface similar to controls, there was a near absence of mature osteoblasts within the suture at all stages. To determine whether these events occur in other sutures, we analyzed immature and mature osteoblasts in the coronal suture at 26 SL (Fig. S4d–f). In *fgfr3*^*lof/lof*^ coronal sutures, a higher number of immature osteoblasts and an absence of mature osteoblasts were found compared to controls, with no significant difference for the immature and mature osteoblasts along the bone surface. We conclude that the same osteoblast maturation abnormalities are present in *fgfr3*^*lof/lof*^ coronal sutures as in the metopic suture.

To understand the higher number of *runx2* and *sp7* positive cells and the absence of *bglap* positive cells within the suture of the *fgfr3*^*lof/lof*^ fish, we first studied cell proliferation rates at 12 and 20 SL by performing balneation EdU experiments. Surprisingly, no differences at either stage were observed (Fig. 4e, g) suggesting that Fgfr3 loss does not impair proliferation of osteoprogenitors and immature osteoblasts within the suture. Subsequently, we investigated apoptosis by performing a TUNEL assay. At 12 SL, we observed no apoptosis for either genotype. However, at 20 and 26 SL, a few randomly localized apoptotic events were observed only within the *fgfr3*^*lof/lof*^ suture (Fig. 4f, h). Given that apoptosis occurs sporadically and at later stages than the observed absence of mature osteoblasts, it is unlikely to be the cause of their disappearance. Instead, this loss is more plausibly explained by an inhibition of immature osteoblast maturation.

All these data demonstrate that the absence of Fgfr3 seems to have a dual effect specifically within the cranial sutures. On one hand, it induces the expansion of osteoprogenitors all along the suture edge, which then differentiate into immature osteoblasts. On the other hand, it inhibits immature osteoblasts maturation, leading to their accumulation at the suture edge.

### Fgfr3 loss is not compensated by other Fgfrs and leads to the overexpression of *fgf18* within the suture

**Fig. 5:**
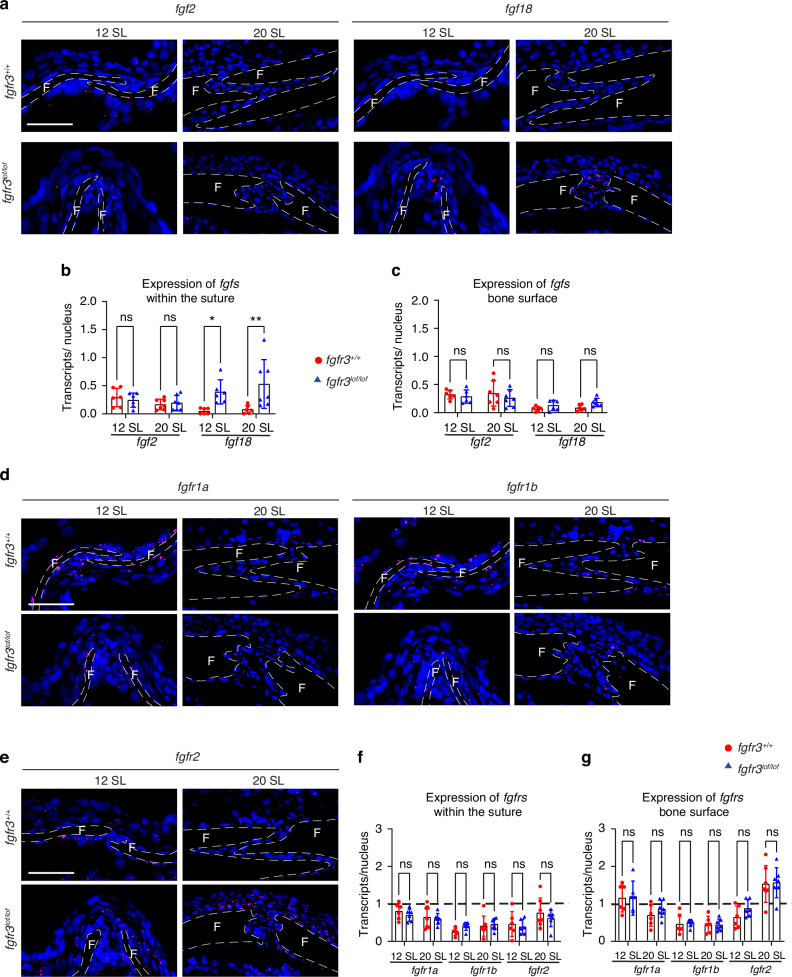
The absence of Fgfr3 is not compensated by other Fgfrs and leads to the overexpression of Fgf18 within the suture. **a** *fgf2* and *fgf18* expression (red) on coronal sections of metopic suture at 12 and 20 SL labeled by RNAscope in situ hybridization. Nuclei were counterstained with DAPI (blue). Scale bar = 50 µm. The dotted lines represent the bone boundaries. RNAscope Quantitative analyses of *fgf2* and *fgf18* expression in *fgfr3*^*+/+*^ versus *fgfr3*^*lof/lof*^ within the suture (**b**) or along the bone surface (**c**) at 12 and 20 SL showed that *fgf18* is significantly more expressed in the *fgfr3*^*lof/lof*^ suture than in *fgfr3*^*+/+*^ suture at 12 and 20 SL (12 and 20 SL: *fgfr3*^*+/+*^ *n* = 6; *fgfr3*^*lof/lof*^ *n* = 6). **d**, **e** *fgfr1a, fgfr1b* and *fgfr2* expression (red) on coronal sections of metopic suture at 12 and 20 SL labeled by RNAscope in situ hybridization. Nuclei were counterstained with DAPI (blue). Scale bar = 50 µm. The dotted lines represent the bone boundaries. RNAscope quantitative analyses of *fgfr1a, fgfr1b* and *fgfr2* expression in *fgfr3*^*+/+*^ versus *fgfr3*^*lof/lof*^ within the suture (**f**) or along the bone surface (**g**) at 12 and 20 SL revealed similar expression of *fgfr1a, fgfr1b*, and *fgfr2* in both genotypes (12 SL: *fgfr3*^*+/+*^ *n* = 6; *fgfr3*^*lof/lof*^ *n* = 6; 20 SL: *fgfr3*^*+/+*^ *n* = 7; *fgfr3*^*lof/lof*^ *n* = 7). Data are presented as mean ± SD. *P*-values were determined by two-way ANOVA: ns: not significant; *P < 0.05; **P < 0.01

The finding that Fgfr3 loss specifically impairs osteoblastogenesis within the suture, while leaving osteoblast formation along the bone surface unaffected, was unexpected, especially considering that *fgfr3* is expressed in both regions (Fig. 1b, c). This differential activity of Fgfr3 may reflect the varying expression levels of its ligands, such as Fgf2 and Fgf18, which are known to be expressed during zebrafish cranial vault development.^28^ Therefore, we examined the expression of *fgf2* and *fgf18* (Fig. 5a). With regard to *fgf2*, no differential expression was observed between the bone surface and the suture, regardless of the genotype at 12 and 20 SL (Fig. 5b, c). However, although *fgf18* expression was low in both the bone surface and suture of *fgfr3*^*+/+*^ fish, it was higher in *fgfr3*^*lof/lof*^ sutures at both stages (Fig. 5b, c). The absence of differential expression of *fgf2* and *fgf18* between the bone surface and suture in *fgfr3*^*+/+*^ fish may suggest that the localized role of Fgfr3 within the suture is not dependent on the expression of these two ligands. However, these results highlight that the absence of Fgfr3 leads to an increase of *fgf18* expression specifically within the suture.

Next to assess if Fgfr3 loss leads to an overexpression of the other *fgfrs* we examined the expression of *fgfr1a*, *fgfr1b* and *fgfr2* (Fig. 5d–g). *fgfr1b* was consistently expressed at low levels during cranial suture formation, with no significant variation based on spatial distribution or genotype. Similarly, *fgfr1a* and *fgfr2* expression did not differ between control and mutant fish. However, *fgfr2* was more highly expressed in the bone surface compared to the suture at 20 SL in both genotypes (Fig. S5). These results revealed, first, that Fgfr3 loss does not lead to an increase in expression of other Fgfrs; second, that other Fgfrs, such as Fgfr2 may play a more prominent role than Fgfr3 at the bone surface.

### Overactivation of canonical Wnt signaling appears as a key contributor to the cranial suture phenotype observed in *fgfr3*^*lof/lof*^ fish

**Fig. 6:**
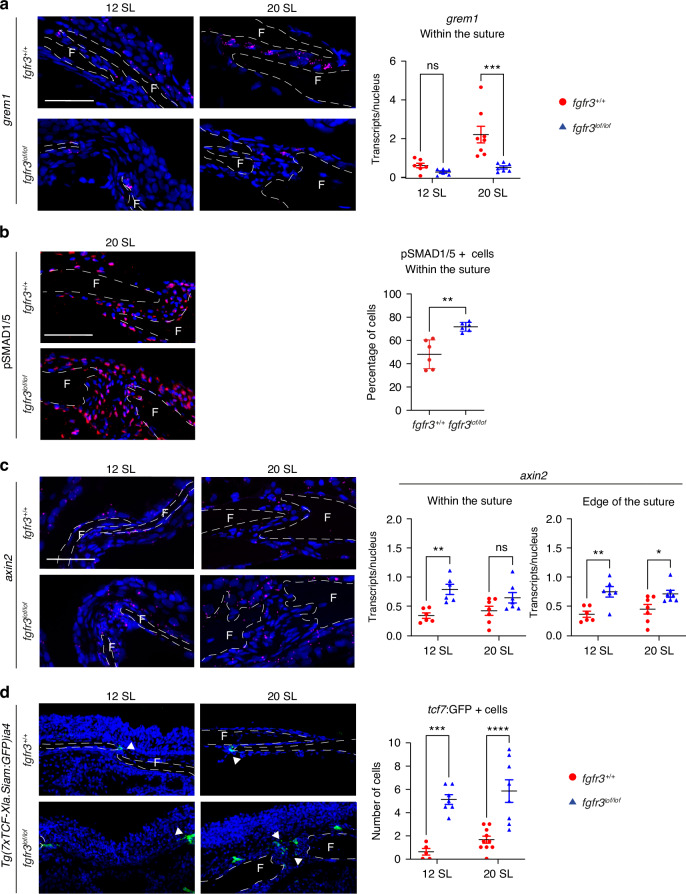
Loss of Fgfr3 modulates actors of the Wnt and BMP pathways. **a** Expression of *grem1* (red) in coronal sections of the metopic suture at 12 and 20 SL, detected by RNAscope. Nuclei are counterstained with DAPI (blue). Scale bar = 50 µm. Quantification shows a significant reduction of *grem1* expression at 20 SL in *fgfr3*^*lof/lof*^ fish. (12 SL: *fgfr3*^*+/+*^ *n* = 6; *fgfr3*^*lof/lof*^ *n* = 6; 20 SL: *fgfr3*^*+/+*^ *n* = 7; *fgfr3*^*lof/lof*^ *n* = 7) *P*-values were determined by two-way ANOVA. **b** Immunofluorescence for pSmad1/5 at the metopic suture, along with quantification of positive cells at 20 SL, revealed an increased proportion of pSmad1/5-positive cells in the mutant sutures suggesting an overactivation of the BMP signaling pathway (*fgfr3*^*+/+*^ *n* = 7; *fgfr3*^*lof/lof*^ *n* = 6). The dotted lines represent the bone boundaries. Scale bar = 50 µm. *P*-values were determined by two-way ANOVA. **c** Expression of *axin2* (red) in coronal sections of the metopic suture at 12 and 20 SL, detected by RNAscope. Nuclei are counterstained with DAPI (blue). Scale bar = 50 µm. Quantification shows that *axin2* expression was increased in *fgfr3*^*lof/lof*^ within the metopic suture compared to controls at 12 SL. Additionally, *fgfr3*^*lof/lof*^ fish showed higher *axin2* expression than controls at both 12 and 20 SL along the metopic suture edge (12 SL: *fgfr3*^*+/+*^ *n* = 6; *fgfr3*^*lof/lof*^ *n* = 6; 20 SL: *fgfr3*^*+/+*^ *n* = 7; *fgfr3*^*lof/lof*^ *n* = 7). *P*-values were determined by two-way ANOVA. **d** Immunofluorescence against GFP (green) was performed on metopic sutures of Tg(7xTCF-Xla.Siam:GFP; *fgfr3*^*+/+*^ or *fgfr3*^*lof/lof*^) fish at 12 and 20 SL. GFP cells within the suture are indicated by white arrowheads Quantification at 12 and 20 SL of the number GFP-positive cells revealed a significantly higher number of positive cells within the suture of *fgfr3*^*lof/lof*^ compared to controls, suggesting an overactivation of the Wnt signaling pathway (12 SL: *fgfr3*^*+/+*^ *n* = 5; *fgfr3*^*lof/lof*^ *n* = 7; 20 SL: *fgfr3*^*+/+*^ *n* = 10; *fgfr3*^*lof/lof*^ *n* = 9)

Next, to further understand the *fgfr3*^*lof/lof*^ suture anomalies, we studied the expression of *prrx1, gli1, axin2*, and *grem1*, genes known to be crucial for suture patency, using RNAscope.^5,11,31^ No significant difference was observed in *prrx1* expression (Fig. S6). For *gli1*, only a slightly higher expression was observed in *fgfr3*^*lof/lof*^ sutures compared to controls at 12 SL, with expression becoming almost absent at 20 SL in both genotypes (Fig. S6), likely reflecting only delayed cranial vault growth. In contrast, *grem1*, which encodes Gremlin1, a BMP antagonist, described as a marker for overlapping bones, showed different patterns.^5^ In controls, its expression was confined to the suture and progressively increased from 12 to 20 SL, whereas in *fgfr3*^*lof/lof*^ sutures, it was limited to the bone tips at 12 SL and significantly reduced by 20 SL (Fig. 6a). To determine whether its downregulation reflects only the absence of bone overlap or also increased activation of the BMP pathway, we quantified phosphorylated SMAD1/5 (pSMAD1/5) within the suture (Fig. 6b). This analysis revealed an increased proportion of pSMAD1/5-positive cells in the absence of Fgfr3 at 20 SL, indicating enhanced BMP signaling.

Moreover, interestingly, significant upregulation of *axin2* in mutants compared to controls were observed at 12 SL within the suture, which appeared to persist over time, although not significantly. Since *axin2* expression seemed higher at the suture edges, where osteoprogenitors and immature osteoblasts reside, we focused our analysis there and found significant higher expression at both 12 and 20 SL in *fgfr3*^*lof/lof*^ suture edge compared to controls (Fig. 6c). Axin2 acts both as a Wnt/β-catenin target gene and a negative regulator of the Wnt canonical pathway, therefore, we next sought to determine whether abnormal activation of the Wnt signaling pathway occurs within *fgfr3*^*lof/lof*^ sutures.^32^ To this end, we used the Wnt reporter line *Tg(7xTCF-Xla.Siam:GFP)*^*ia4*^ to generate *Tg(7xTCF-Xla.Siam:GFP)*^*ia4*^*; fgfr3*^*lof/lof or +/+*^ and examined GFP-positive cells at the metopic suture at 12 and 20 SL. GFP positive cells were localized at the tips of the frontal bones in both genotypes at 12 SL but a greater number of positive cells were present in mutant compared to controls. At 20 SL, GFP-positive cells remained elevated and extended along the suture in mutants, while staying restricted to bone tips in controls, indicating increased canonical Wnt activation (Fig. 6d).

**Fig. 7:**
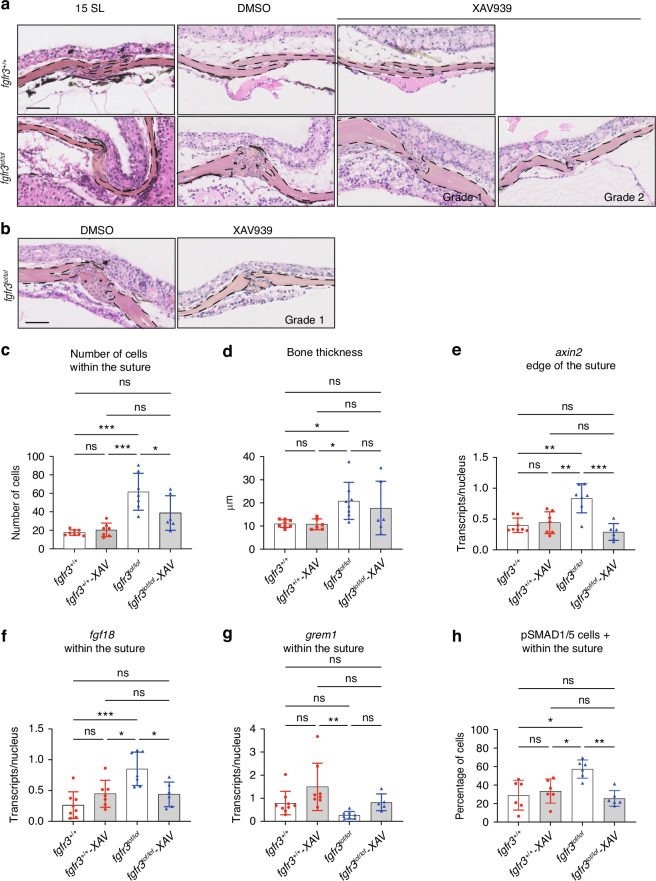
Overactivation of canonical Wnt signaling appears as a key contributor to the cranial suture phenotype observed in *fgfr3* fish. *lof/lof* (**a**, **b**) HE stained coronal sections at the metopic suture level of the zebrafish head (*fgfr3*^*+/+*^ or *fgfr3*^*lof/lof*^) treated with DMSO or 2.5 µmol/L XAV939 demonstrated in a that the inhibition of canonical Wnt signaling partially rescue the suture morphology of the *fgfr3*^*lof/lof*^ fish with reduced interdigitation and fewer intersutural cells (rescue grade 1), and in one case, a tendency for overlapping bones (rescue grade 2). (*fgfr3*^*+/+*^ DMSO *n* = 8; *fgfr3*^*lof/lof*^ DMSO *n* = 7; *fgfr3*^*+/+*^ XAV939 *n* = 7; *fgfr3*^*lof/lof*^ XAV939 *n* = 6). Scale bar = 50 µm. **b** shows that Wormian bones (indicated by black asterisks) in *fgfr3*^*lof/lof*^ fish persist after XAV939 treatment. **c** Quantification of the number of cells within the suture (*fgfr3*^*+/+*^ DMSO *n* = 8; *fgfr3*^*+/+*^ XAV939 *n* = 7, *fgfr3*^*lof/lof*^ DMSO *n* = 7; *fgfr3*^*lof/lof*^ XAV939 *n* = 6) showed that the *fgfr3*^*lof/lof*^ XAV939 fish present a lower number of cells within the suture compared to *fgfr3*^*lof/lof*^ DMSO fish. **d** Quantification of the bone thickness within the suture (*fgfr3*^*+/+*^ DMSO *n* = 8; *fgfr3*^*lof/lof*^ DMSO *n* = 7; *fgfr3*^*+/+*^ XAV939 *n* = 7; *fgfr3*^*lof/lof*^ XAV939 *n* = 6) showed that the *fgfr3*^*lof/lof*^ XAV939 fish present a slight and variable reduction of bone thickness compared to *fgfr3*^*lof/lof*^ DMSO fish. **e**–**g** Quantitative RNAscope analysis revealed decreased expression of *axin2* at the edge of the suture and *fgf18* within the suture in *fgfr3*^*lof/lof*^ XAV939 fish compared to *fgfr3*^*lof/lof*^ DMSO fish (*fgfr3*^*+/+*^ DMSO *n* = 8; *fgfr3*^*+/+*^ XAV939 *n* = 7 *fgfr3*^*lof/lof*^ DMSO *n* = 7; *fgfr3*^*lof/lof*^ XAV939 *n* = 6). *grem1* expression showed a slight increase of its expression within the suture of *fgfr3*^*lof/lof*^ XAV939 fish compared to *fgfr3*^*lof/lof*^ DMSO fish (*fgfr3*^*+/+*^ DMSO *n* = 8; *fgfr3*^*lof/lof*^ DMSO *n* = 7; *fgfr3*^*+/+*^ XAV939 *n* = 7; *fgfr3*^*lof/lof*^ XAV939 *n* = 5). **h** Quantification of pSMAD1/5 positive cells, revealed lower proportion of pSmad1/5-positive cells in the *fgfr3*^*lof/lof*^ XAV939 fish compared to *fgfr3*^*lof/lof*^ DMSO fish (*fgfr3*^*+/+*^ DMSO *n* = 6; *fgfr3*^*lof/lof*^ DMSO *n* = 6; *fgfr3*^*+/+*^ XAV939 *n* = 6; *fgfr3*^*lof/lof*^ XAV939 *n* = 6). *P*-values were determined by Student’s *t*-test: ns = not significant; *P < 0.05; **P < 0.01; ***P < 0.001; ****P < 0.000 1. Data are presented as mean ± SD

To confirm this hypothesis, *fgfr3*^*+/+*^ and *fgfr3*^*lof/lof*^ fish were treated by balneation with the β-catenin inhibitor XAV939 (2.5 μmol/L) or 0.1% DMSO for 1 month, starting at 15 SL, the stage at which the frontal bones in *fgfr3*^*lof/lof*^ fish are closed (Fig. 7a). Histological analysis of transverse sections of the metopic suture revealed that DMSO treatment had no observable effect on the suture structure of *fgfr3*^*lof/lof*^ fish, whereas treatment with XAV939 led to notable improvements in cranial suture morphology. Most treated mutants displayed reduced interdigitation and fewer intersutural cells (rescue grade 1), and in one case, a tendency for overlapping bones was observed (rescue grade 2), suggesting a partial restoration of normal suture architecture. Notably, Wormian bones persist in *fgfr3*^*lof/lof*^ fish despite XAV939 treatment (Fig. 7b). To substantiate this partial phenotypic rescue, we quantified both the number of intersutural cells and bone thickness. XAV939-treated *fgfr3*^*lof/lof*^ fish showed a significant reduction, with cell numbers no longer differing significantly from controls, unlike DMSO-treated mutants (Fig. 7c). The effect of XAV939 on bone thickness appears weaker, likely due to the variability observed (Fig. 7d). To further assess the molecular impact of XAV939 treatment, we analyzed the expression of *axin2, fgf18*, and *grem1* by RNAscope (Fig. 7e–g). In *fgfr3*^*lof/lof*^ fish, *axin2* expression at the suture edges was normalized following XAV939 treatment, confirming suppression of Wnt/β-catenin pathway overactivation. Notably, the elevated *fgf18* expression previously observed in *fgfr3*^*lof/lof*^ fish was also restored to control levels upon treatment. As for *grem1*, its expression was slightly increased in XAV939-treated *fgfr3*^*lof/lof*^ fish compared to DMSO-treated mutants. Finally, we assessed the effect of XAV939 treatment on the number of pSMAD1/5-positive cells within the sutures and found that inhibition of the Wnt/β-catenin pathway during cranial suture formation in *fgfr3*^*lof/lof*^ fish led to a significant reduction in the proportion of pSMAD1/5-positive cells compared to DMSO-treated mutants (Fig. 7h). Notably, their numbers no longer differed significantly from control levels.

Altogether, these results highlight a key role for the canonical Wnt signaling in the cranial suture phenotype of *fgfr3*^*lof/lof*^ fish and suggest regulatory crosstalk between Fgfr3, canonical Wnt pathway, Fgf18, and potentially the BMP pathway.

## Discussion

**Fig. 8:**
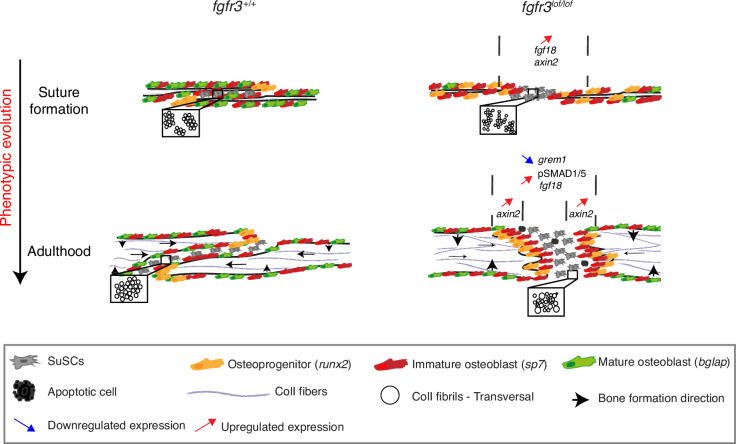
Impact of Fgfr3 absence on cranial suture formation. The absence of Fgfr3 exerts pleiotropic effects on cranial suture formation, leading to significant structural defects. Without Fgfr3, proper overlap of the sutures is prevented, resulting in bone widening. These structural abnormalities are associated with altered osteogenesis, as Fgfr3 deficiency disrupts the expansion of osteoprogenitors and inhibits osteoblast maturation within the suture, which could consequently slow longitudinal bone growth. Additionally, the absence of Fgfr3 affects the extracellular matrix (ECM) at the ends of bones and within the suture itself. From a signaling pathway perspective, the loss of Fgfr3 triggers the overexpression of Fgf18 and causes the misregulation of *axin2* and *grem1* expression and leads to an increase of Smad1/5 phosphorylation. This positions Fgfr3 as a central regulator of cranial suture formation in a complex network involving FGF, canonical Wnt, and potentially BMP signaling pathways

By investigating cranial suture development from initiation to adulthood of our Fgfr3 LoF fish model we demonstrated the multifaceted involvement of Fgfr3 in suture biology (Fig. 8).^28^ We provide evidence that Fgfr3 is crucial for ensuring proper fibrillogenesis within the suture, establishing a correctly formed OF by limiting the number of osteoprogenitors at the bone ends, and promoting the maturation of osteoblasts specifically at the suture edge. We demonstrated that Fgfr3 regulates cranial suture formation via the canonical Wnt pathway and possibly the BMP pathway, while also highlighting a potential regulatory loop involving *fgf18* expression. These results are essential as until now, the involvement of FGFR3 in cranial suture formation had only been illustrated by the existence of the craniosynostoses associated with FGFR3 mutations (Muenke and CAN syndrome) and a few studies reporting occasional premature fusion of coronal sutures in mouse models expressing *Fgfr3* GoF mutations.^19,20,23,33^

Our study focuses on the late stages of cranial vault development, when the bones converge and the suture forms. In this phase, periosteal osteoblasts drive skull widening and remodeling, while suture osteoblasts are responsible for maintaining continuous cranial expansion and keeping the suture open. Analysis of osteogenesis at both sites using *Tg(runx2:*GFP*; fgfr3*^*lof/lof or +/+*^*)*, and *Tg(sp7:*mCherry*; bglap:*GFP*; fgfr3*^*lof/lof or +/+*^*)* lines showed that Fgfr3 loss does not affect osteogenesis along the bone surface. In contrast, from the suture initiation onwards, at bone extremities, Fgfr3 absence increases the number of osteoprogenitors (Runx2: GFP positive cells), which are no longer confined to bone tips, leading to an expanded ossification front over time. Interestingly, this increase of Runx2 positive cells associated with the lack of bone overlap has been described in *sp7*^-/-^ fish.^34^ In that study, the authors linked the increase in Runx2 positive cells to the absence of *sp7* expression, which is essential for the normal progression of osteoblast differentiation. In contrast, in *fgfr3*^*lof/lof*^ fish, differentiation appears blocked not at the transition from osteoprogenitors to immature osteoblasts but from immature to mature osteoblasts. This is evidenced by the higher number of *sp7:* mCherry positive cells and the absence of *bglap:*GFP postive cells at the suture edge, unrelated to apoptosis. These findings showed that Fgfr3 positively regulates osteoblast maturation during suture formation, as we demonstrated at an earlier stage of cranial bone development.^28^ However, the increase in *runx2-* and *sp7*-positive cells remains complex to interpret, as no increase in osteoprogenitor or immature osteoblast proliferation was detected at the suture edge. Instead, it may result from enhanced recruitment of osteoprogenitors from differentiating SuSCs and/or the accumulation of cells that fail to mature. In any case, the rearrangement of osteogenic cells specifically at the bone extremities, but not along the bone surface, forming what can be termed an extended OF, likely underlies: (1) the formation of sutures with abutting rather than overlapping bones, (2) the progressive widening of the bone extremities, and (3) part of the increased cellularity observed within the suture.

Our data reveals, that the absence of Fgfr3 appears to lead to overactivation of the Wnt signaling pathway at the suture edges, as evidenced by both the overexpression of *axin2*, a Wnt/β-catenin target gene and the increased number of *7xTCF-Xla.Siam:*GFP-positive cells in Fgfr3 deficient fish. Moreover, Wnt pathway overactivation appears to be a major contributor to the cranial suture anomalies observed in *fgfr3*^*lof/lof*^ fish, as inhibition of the canonical Wnt pathway with XAV939 treatment partially rescues this phenotype. This data suggests that Fgfr3 may act as a potential inhibitor of the canonical Wnt signaling pathway. Notably, Fgfr3 has already been described as such during chondrogenesis, specifically in pharyngeal skeleton formation in zebrafish.^27^ In addition, recent work by Bobzin et al. highlighted crosstalk between FGF and Wnt/β-catenin pathways in sutures of mice, demonstrating that FGFR2 activation at the OF of the frontal bones regulates anterior fontanel closure by inhibiting Wnt signaling through Wif1.^35^ Within the suture, Wnt signaling is involved in regulating the number of SuSCs and also osteoblast differentiation.^36^ Aberrant β-catenin activation has been shown to widen all cranial sutures in mice, accompanied by increased *Runx2* and *Sp7* expression, the absence of the mature osteoblast marker *Osteopontin*, and enhanced BMP signaling.^37^ These findings align with our observations and further support that Fgfr3 might promote maturation of osteoblasts via the inhibition of canonical Wnt signaling in cranial sutures. Interestingly, we noted in *fgfr3*^*lof/lof*^ an overexpression of *fgf18*, one of the Fgfr3 ligands essential for cranial vault development.^38^ Notably*, Fgf18* is a direct Wnt target gene, regulated positively via a TCF/Lef binding site, in cooperation with RUNX2.^39^ The restoration of Fgf18 expression to normal levels in *fgfr3*^*lof/lof*^ sutures following XAV939 treatment strongly supports the notion that canonical Wnt pathway activation is responsible for the upregulation of *fgf18* in the absence of Fgfr3.

Additionally, we showed that loss of Fgfr3 leads to both a significant decrease in *grem1* expression and to an increase in the number of pSMAD1/5 positive cells within the sutures. Grem1 is an antagonist of the BMP signaling pathway recently identified as a marker of a SuSC subpopulation in zebrafish.^5^ Interestingly, Farmer et al., reported that the absence of *grem1* in zebrafish causes a slight delay of cranial vault formation. However, when Grem1 is absent along with Nog2 and Nog3, two other BMP antagonists, it leads to overactivation of BMP signaling in the suture, resulting in the same suture anomalies seen in our *fgfr3*^*lof/lof*^ model (e.g., failure of bone overlaps and the presence of ectopic bone). This phenotypic similarity, combined with decreased *grem1* expression and the increase number of pSMAD1/5 positive cells in *fgfr3*^*lof/lof*^ metopic sutures, suggests that the absence of Fgfr3 may increase BMP signaling within the suture. Given that the BMP pathway promotes osteogenesis, its activation in the absence of Fgfr3 may enhance osteoprogenitor recruitment along the suture. This notion is reinforced by the observation of BMP pathway activation in *sp7*^⁻/⁻^ zebrafish, which display also elevated numbers of Runx2-positive cells.^34^ Additional experiments are required to validate this hypothesis and to elucidate the link between Fgfr3 loss and decreased *grem1* expression. Existing data from mouse models, on cranial sutures suggest the opposite that FGF signaling promotes BMP signaling.^40,41^ Additionally, it was reported that FGF18 modulates *Grem1* expression via the activation of FGFR3 in chondrocytes located in synchondroses or in articular cartilage.^42,43^ Although regulation of Grem1 by Fgf18 in cranial sutures has not been documented, it is plausible that Fgf18 regulates *grem1* expression through binding to other Fgfrs. At the RNA level, we did not observe an increase in *fgfr1a*, *fgfr1b*, and *fgfr2* expression within the suture in the absence of Fgfr3, suggesting the absence of a compensatory mechanism. Nevertheless, since *fgfr1a, fgfr1b*, and *fgfr2* are expressed within the suture, albeit to a lesser degree than Fgfr3, their activation through Fgf18 binding remains a possibility. Interestingly, we observed that inhibition of the canonical Wnt signaling pathway induces a slight increase in *grem1* expression and a significant decrease of pSMAD1/5 positive cells in *fgfr3*^*lof/lof*^ fish. This suggests that BMP signaling could be inhibited either directly through canonical Wnt pathway inhibition and/ or indirectly via decreased *fgf18* expression, which modulates *grem1* expression. Further studies are needed to understand this mechanism.

Finally, our work provides evidence that the absence of Fgfr3 may impact osteogenesis not only through direct pathway activation but also by altering the microenvironment of the cells, particularly the collagen network, which, by transmitting biomechanical signals influences cell fate. In our previous study, we showed that Fgfr3 loss leads to the overexpression of ECM-regulating proteins like *col6a1, col12a1a*, and *col12a1b*.^28^ ColXII is known to bind ColI fibrils, regulating their maturation and organization, and thereby helping to absorb biomechanical stress.^15,44,45^ We show here that the loss of Fgfr3 disrupts the organization of ColI fibrils in the bones near the suture and within the suture itself, due to both a delay in cranial suture formation and a modification of its shape. This is accompanied by a fibrillogenesis defect, potentially linked to ColXII overexpression. We suggest that modifications in the osteogenic cell environment, including collagen network remodeling and the predominance of lateral over longitudinal bone growth, may alter the mechanical forces exerted on osteogenic cells within the suture. These changes could influence cell fate and potentially explain the progressive emergence of random apoptosis, an as-yet unexplained phenomenon.

In conclusion, this study identifies Fgfr3 as a key modulator of cranial suture homeostasis, acting through complex interactions with Fgf18, the canonical Wnt pathway, and potentially the BMP signaling pathway. Together, these findings provide new insights into suture homeostasis and FGFR3-related craniosynostoses, paving the way for the development of targeted therapeutic strategies.

## Materials and methods

### Zebrafish husbandry, fgfr3^lof^ fish and transgenic lines and genotyping

Zebrafish were raised, hatched and maintained in an aquatic facility at 28 °C (approval number 2018080216094268 and APAFIS #36172-2022041412092374 v1), in accordance with European Directive 2010/63/EU for animals. At the juvenile stage, during cranial bone formation (approximately 1–2 months of age), developmental stages were determined using the standard length (SL) of the fish, measured from the snout to the caudal peduncle^46^. For the analyses performed at the adult stage, and in order to maintain the same unit of measurement, we defined 3-month-old fish as corresponding to 20 SL and 6-month-old fish to 26 SL, based on the size of wild-type fish. *fgfr3*^*lof*^ fish correspond to the *fgfr3*^*lof1*^ line described previously and were genotyped as previously described.^28^ Transgenic lines *Tg(runX2:GFP), Tg(sp7:mCherry), Tg(bglap:GFP)*^46^ and *Tg(7xTCF-Xla.Siam:GFP)*^*ia4*^^47^ labeling respectively osteoprogenitors, immature osteoblasts, mature osteoblasts and activation of Wnt signaling have been previously described.

### Histological analyses

Zebrafish at 12, 20 and 26 SL were fixed for 24 h in 4% paraformaldehyde at 4 °C, the heads were decalcified in a 0.5 mol/L EDTA solution pH8 for 1–5 days, then embedded in paraffin. Paraformaldehyde-Fixed and Paraffin-Embedded (FFPE) (4 µm) sections were cut either transversally or sagittally. Haematoxylin Eosin (HE) and Sirius red-Alcian blue staining were performed after dewaxing with Xylene substitute, Neo-Clear® followed by rehydration with decreasing ethanol baths. For HE staining, the sections were incubated sequentially with Haematoxylin for 30 s, LiCO_3_ for 2 s and Eosin for 10 s. For Sirius Red-Alcian blue staining, sections were incubated in Weigert’s solution (Hematein 0.1 g/mL in EtOH 95° and FeCl_3_ 29% HCl 37% v/v) for 5 min, washed with acetic acid 1%, incubated 30 min with Alcian blue (1 mg/mL; 10% HCl 1 N), and finally treated with 0.1% direct red diluted in aqueous saturated picric acid for 2 h. Image acquisitions were performed using an Olympus IX81 microscope equipped with an Olympus IX2-UCB camera. Quantification of bone thickness was performed on the two bones of one section per individual using the Fiji software (ImageJ).

### Immunohistochemistry (IHC)

Zebrafish at 12, 20, and 26 SL were fixed for 24 h in 4% paraformaldehyde at 4 °C, heads were decalcified for 1–5 days in a 0.5 mol/L EDTA solution pH8 and impregnated in sucrose 30% during at least 10 days, then, embedded in frozen medium containing 30% sucrose and 7% gelatine. 20 µm sections were cut in transverse orientations. The polyclonal primary antibodies used are anti-Collagen I (ColIα1, Abcam, ab23730), anti-collagen VI (ColVIα1^48^), anti-zebrafish collagen XII antibodies (ColXIIα1^49^), anti-mCherry (Takara, 632496) and anti-GFP (Invitrogen; A11122). Blocking and incubation protocols varied by antibody: For Col I, sections were washed with PBSTr, blocked in BB1 (4% BSA, 0.1% Triton-X100), then incubated overnight with primary antibody (1:200) in BB1 at 4 °C. For Col XII, mCherry, and GFP, sections were blocked in BB2 (3% sheep serum, 1% BSA), incubated 1 h with primary antibody (1:250) in BB2 at RT. For Col VI, sections were blocked in BB3 (5% goat serum), incubated overnight with primary antibody (1:400 in BB3) at 4°. Then After several wash in PBSTr the final steps of IHC for whole labeling consist of a 1 h incubation with an Alexa fluor^TM^ goat anti-rabbit 594 or 647 (Invitrogen) diluted 1:500 in PBS followed by three 5-min washes with PBS. IHC anti-pSMAD1/5 were performed following the protocol of Farmer et al. on FFPE sections using the antigen retrieval reagents in the RNAscope Fluorescent Multiplex kit (ACD), slides were blocked for 1 h in 10% goat serum, stained with primary overnight (1:100, Cell Signaling #9516) and stained for 1 h in 5% goat serum with secondary (1:250, Alexa fluor^TM^ goat anti-rabbit 594, Invitrogen).^5^ All the slides were mounted in DAPI Fluoromount-G ^TM^ (Electron Microscopy Sciences—#17984-24). IHC were imaged with a Spinning Disk Zeiss controlled by Zen Blue software (Zeiss, Jena, Germany) and processed on the Fiji software (ImageJ). The quantification represents the ratio between the number of positively labeled cells and the number of DAPI-stained cells.

### EdU and TUNEL labeling

For EdU analyses, 12 and 20 SL *Tg(runx2 :*GFP*)* fish were immersed in an EdU solution at 12.5 µg/mL during 48 hpf. EdU incorporation was subsequently detected on frozen sections (30% sucrose, 7% gelatin) for 12 SL fish and on FFPE sections for 20 SL fish. The EdU was detected using the EdU Click-iT Plus EdU Alexa Fluor 647 Imaging kit (Life Technologies, Carlsbad, CA, USA), following the manufacturer’s protocol. TUNEL assays were performed on the FFPE sections using the DeadEnd Fluorometric TUNEL system (Promega, Madison, WI, USA), following the manufacturer’s instructions (permeabilization step with proteinase K at 10 mg/mL diluted in a PBS at 1:500 for 7 min). Images were captured with a Spinning Disk Zeiss controlled by Zen Blue software (Zeiss, Jena, Germany) and processed using the Fiji software (ImageJ). The TUNEL experiment was quantified at 20 and 26 SL on two adjacent sections per individual. At 12 SL, TUNEL experiment was performed only on *n* = 3 per genotype due to the absence of positive cells.

### RNAscope assay

RNAscope Assays were performed on FFPE sections according to the manufacturer’s instructions « RNAscope Multiplex Fluorescent Reagent Kit v2 Assay » provided by ACD a bio-techne brand. After dewaxing, the slides were incubated with Target Retrieval and Protease Plus reagents for 15 min and 30 min, respectively. The zebrafish *fgfr1a* (C1-409441); *fgfr1b* (C2-409451); *fgfr2* (C1-420961); *fgfr3* (C1-438971); *fgf2* (C1-420951); *fgf8a* (C2-559351); *fgf18* (C3-123572); *axin2* (C1-465351); *gli1* (C2-542721); *prrx1* (C3-535321); *grem1a* (C1-535291) probes were designed and synthesized by the manufacturer. The universal negative control with probes targeting dapB gene (320871) provided by ACD was used as reference. Double labeling was performed following the manufacturer’s instructions. The probes were incubated for 2 h at 40 °C, with a dilution of 1/50 for each probe. Opal 570 (Akoya, Cat. No. FP1488001KT) and Opal 650 (Akoya, Cat. No. FP1496001KT) each diluted to 1:500 were used to reveal the C1 and C2 or C3 probes, respectively. The slides were mounted using ProLong^TM^ Gold antifade reagent (Invitrogen). These experiments were imaged with a Spinning Disk Zeiss controlled by Zen Blue software (Zeiss, Jena, Germany) and processed on the Fiji software (ImageJ). Quantification was based on the ratio of mRNA dots (RNAscope signals) to DAPI-stained nuclei across two adjacent sections. For sutural analysis, all suture cells were counted in *fgfr3*^*+/+*^ fish, while in *fgfr3*^*lof/lof*^ fish, only cells adjacent to bone edges were included, based on transgene expression (*runx2:GFP, sp7:mCherry, bglap:GFP*). Quantification along bone surfaces included cells bordering the frontal bones.

### Polarization-resolved second harmonic generation microscopy

Multiphoton images of unstained FFPE sections of *fgfr3*^*+/+*^ and *fgfr3*^*lof/lof*^ fish at 12 and 26 SL stages were acquired using a custom-built upright laser scanning setup, as previously described.^50^ Laser excitation was tuned to 860 nm and excitation power was 2–8 mW at the sample using a 25×, NA 1.05 water immersion objective (XLPLN-MP, Olympus). Both two-photon excited fluorescence (2PEF) and SHG signals were detected in parallel in two trans-detection channels. Endogenous 2PEF enabled to visualize the tissue morphology, while SHG was obtained specifically from collagen fibrils. Polarization-resolved SHG imaging (P-SHG) was performed by recording series of images excited by linear polarizations rotated from 0° to 170° in 10° steps.^50^ Z-stacks of such P-SHG images were recorded at 200 kHz with an axial step of 0.5 µm and a pixel size of 420 nm. Automated processing by custom written Matlab code then provided: (i) the 2PEF and SHG images averaged over all excitation polarizations, which mitigates the polarization sensitivity; (ii) a pixel-scale orientation map of collagen fiber orientation within the imaging plane.^50^ The latter map was obtained after applying a 2 × 2 binning filter to increase the signal-to-noise ratio. Pixels with a coefficient of determination less than 0.5 were filtered out.^50^

Two quantitative parameters were then calculated from these data, keeping only the image from each z-stack that had the highest mean SHG signal. First, the collagen density within the frontal bone was estimated by calculating the mean intensity of the SHG image normalized to the square of the excitation power. Second, the orientation order of the collagen fibers was quantified by computing the circular standard deviation of the orientation distribution obtained from P-SHG images. This disorder metric assesses the width of the orientation distribution using circular statistics; it increases when the orientation disorder increases. To account for the variation of overall orientation of the bone that would bias a measure of absolute local orientation, this circular standard deviation was computed using the P-SHG orientation relative to the orientation of the bone structure, by computing the direction of the frontal bone locally and eliminating small outgrowths.

### Transmission electron microscopy

Calvaria from zebrafish at 9, 12, and 20 SL were dissected inside the fixation buffer (for 50 mL: 10 mL of cacodylate 0.5 mol/L, 75 µL of glutaraldehyde, 12.5 mL of PFA 4% qsp 50 mL H_2_O). The calvarias were left at least 5 days inside the fixation buffer at 4 °C and washed 3 times 15 min in PBS, then decalcified 24 h in EDTA 0.5 mol/L pH8 at 4 °C. Samples were post fixed in 1% osmium tetroxide 0.1 mol/L (Electron Microscopy Science, UK) in 0.1 mol/L Phosphate Buffer (PB) (pH 7.4). Samples were washed 3 times in H_2_O then dehydrated in alcohol grades: 70% Ethanol 10 min, 90% Ethanol 10 min, 100% Ethanol 3 × 15 min, 100% Propylene oxide (Electron Microscopy Science, UK) 2 × 5 min. Resin infiltration was performed as following: mix 1:1 Epikote 812: propylene oxide 30 min followed by mix 1:2 Epikote 812: propylene oxide overnight room temperature. Samples were washed in 100% Epikote 812 then embedded in silicon molds in 100% Epikote 812, and Polymerized in a 60 °C oven for 24 h. Ultrathin sections were cut at 90 nm with a Leica UFC7 ultramicrotome (Leica Microsystems GmbH, Germany) and deposed on Gilder grids 200 mesh (Electron Microscopy Science, UK). They were counterstained with uranyl acetate 7% (LFG, France) and Reynold’s lead citrate (LFG, France). Samples were examined in a JEOL 1011 transmission electron microscope (JEOL, Japan) with an ORIUS 1000 CCD camera (GATAN, France), operated with Digital Micrograph software (GATAN, France) for acquisition.

### Drug treatment

XAV939 was purchased from Sigma-Aldrich, St. Louis, MO, USA (reference X3004) and was dissolved with DMSO to get a stock solution of 16 mmol/L. *fgfr3*^*+/+*^ and *fgfr3*^*lof/lof*^ were treated by balneation with 2.5 µmol/L XAV939 or 0.1% DMSO from 15 SL during 1 month. The fish were isolated by 3–5 fish in a volume of 500 mL. The water was changed every 2 days.

### Statistical analysis

All statistical analyses were performed using GraphPad Prism (version 10, GraphPad Software, La Jolla, CA, USA). Data normality and lognormality were assessed using the D’Agostino & Pearson, Anderson-Darling, Shapiro-Wilk, and Kolmogorov-Smirnov tests. For data following a normal distribution, statistical comparisons were performed using an unpaired *t*-test when analyzing an independent variable between two groups. A two-way ANOVA was used to assess statistically significant differences between three or more independent groups split across two variables. Non-normally distributed data were analyzed using a non-parametric Mann-Whitney test for two-group comparisons. A significance threshold of *P* < 0.05 was applied to all statistical tests. *P*-values reported in figure legends were considered statistically significant as follows: *, *P* < 0.05; **, *P* < 0.01; ***, *P* < 0.00 1; and ****, *P* < 0.000 1. All values are presented as mean ± SD.

## Supplementary information

Supplementary information

## Data Availability

The data sets generated and/or analyzed during the current study are available upon reasonable request from the lead contact, E.D. (emilie.dambroise@inserm.fr).
