# Supplementary material for: Fgfr3–Wnt signaling crosstalk is involved in maintaining cranial suture integrity
Source: Bone Res. 2026 Jul 30;14:76. doi: 10.1038/s41413-026-00558-w (PMC13421612; doi:10.1038/s41413-026-00558-w)
Supplement: Supplementary file 1 — Supplementary information [file 41413_2026_558_MOESM1_ESM.docx]

**Fgfr3–Wnt signaling crosstalk is involved in maintaining cranial suture integrity.**

corresponding author:

Dr Dambroise Emilie

[emilie.dambroise@inserm.fr](mailto:emilie.dambroise@inserm.fr)

tel: 0033142754250

**Supplementary information**

**Figure S1:** Fgfr3 is required for coronal suture formation.

**Figure S2:** Fgfr3 loss has no impact on osteoclast activity within the suture.

**Figure S3:** Fgfr3 is an inhibitor of osteoprogenitors expansion within the suture.

**Figure S4:** Fgfr3 loss alters osteoblast maturation across coronal and metopic sutures in adulthood.

**Figure S5:** *fgfr2* shows elevated expression at the bone surface.

**Figure S6:** *gli1* and *prrx1* expression during cranial suture formation.


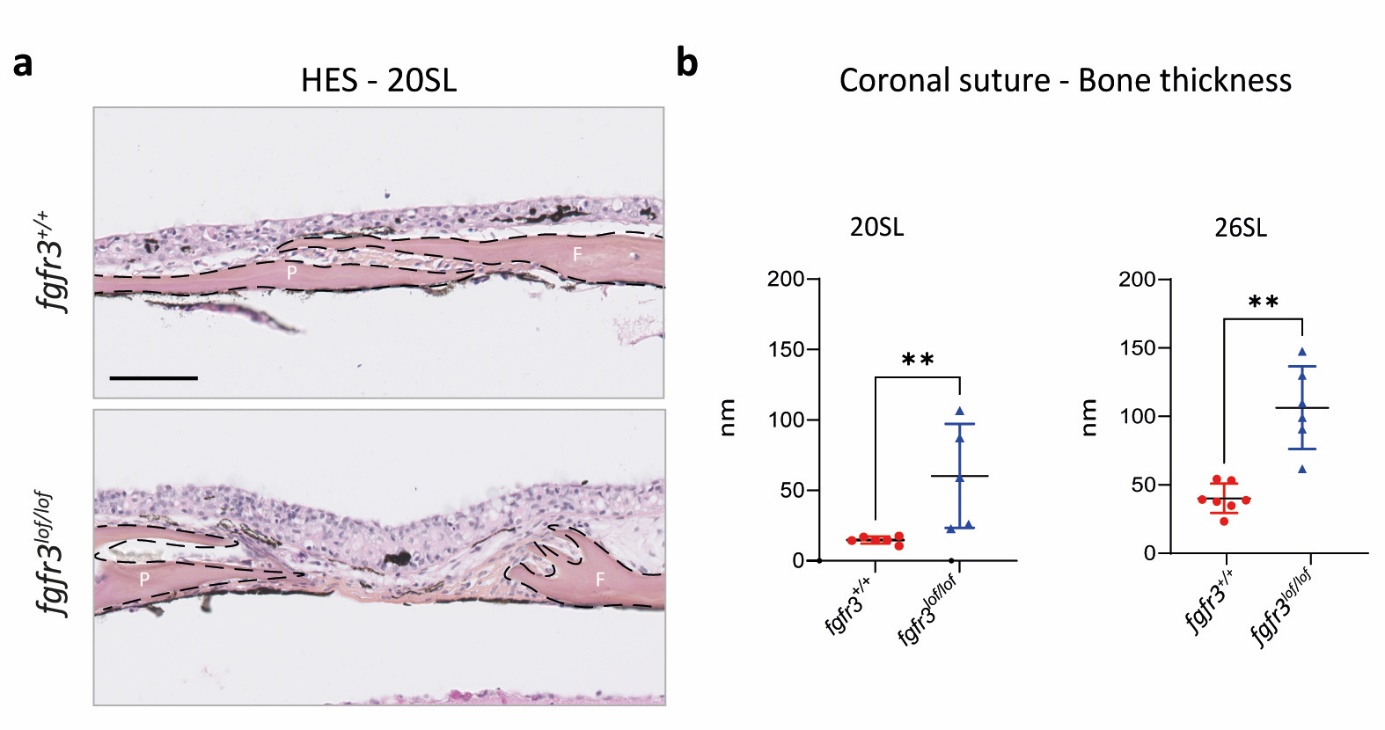


**Figure S1. Fgfr3 is required for coronal suture formation**. (a) HE stained sagittal sections of the zebrafish head at the coronal suture level at 20 SL demonstrated that the absence of Fgfr3 prevents bones overlap and coronal suture formation. The dotted lines represent the bone boundaries. Scale bar = 50 μm. (b) Measurement of bone thickness near the coronal suture showed that the *fgfr3^lof/lof^* bones are thicker than *fgfr3+/+* bones at the adult stages 20 SL (*fgfr3^+/+^* n=6; *fgfr3^lof/lof^* n=6) and 26 SL (*fgfr3^+/+^* n=7; *fgfr3^lof/lof^* n=6). F: frontal bone. P: Parietal bone. Data are presented as mean ± SD. The p-value were determined by Student’s t tests: ns: not significant; p < 0.05 = *; p < 0.01 = **; p < 0.001 = ***; p < 0.0001 = ****.


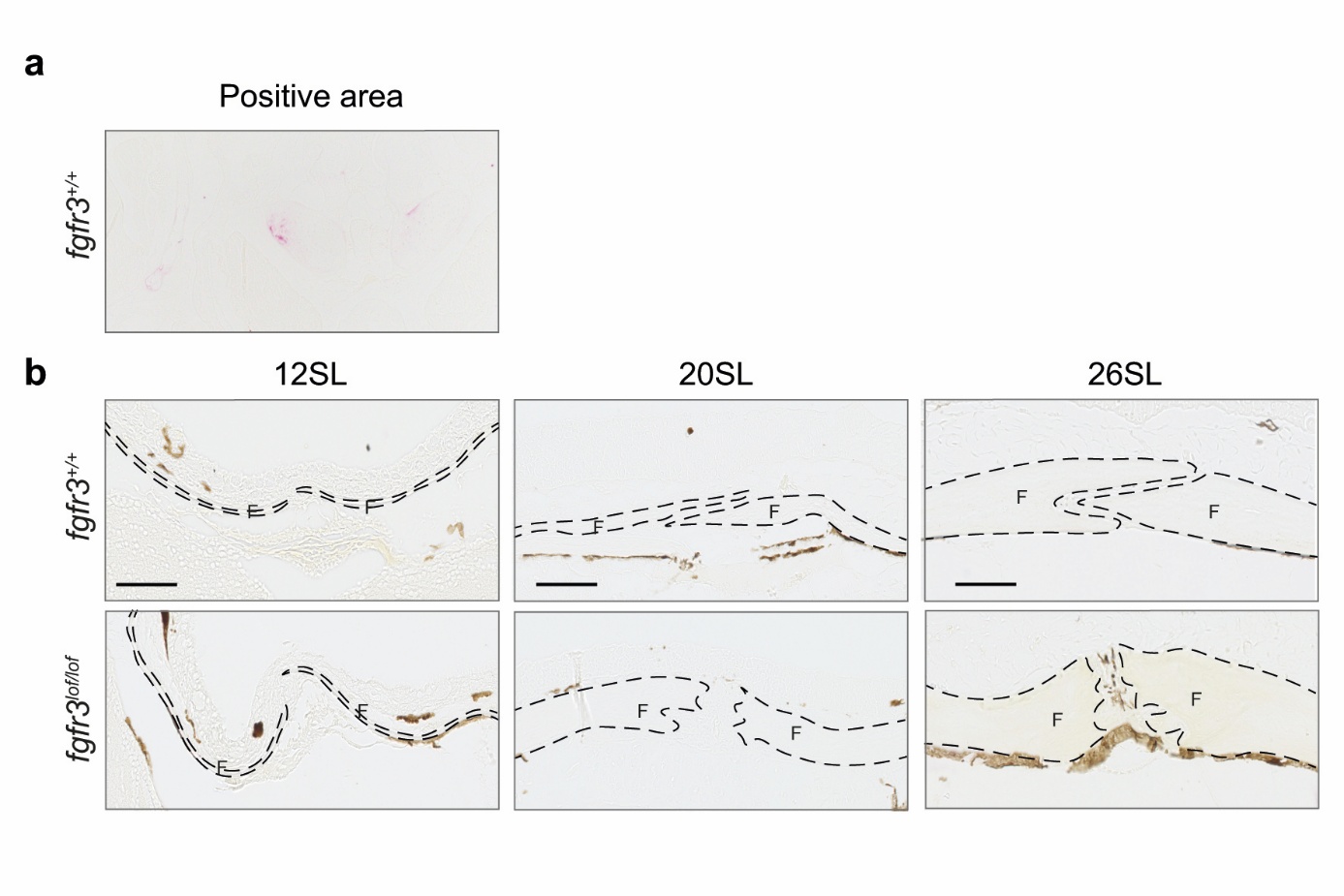


**Figure S2. Fgfr3 loss has no impact on osteoclast activity within the suture.** TRAP-stained sagittal sections of the zebrafish head revealed that TRAP positive cells are present on the viscerocranium (a) and the absence of TRAP positive cells within the metopic suture (b) in both *fgfr3^lof/lof^* and *fgfr3*^+/+^ zebrafish at 12, 20 and 26 SL (12, 20 and 26 SL *fgfr3^+/+^* n=3; *fgfr3^lof/lof^* n=3). The dotted lines represent the bone boundaries. F: Frontal bone. Scale bar = 50 μm.


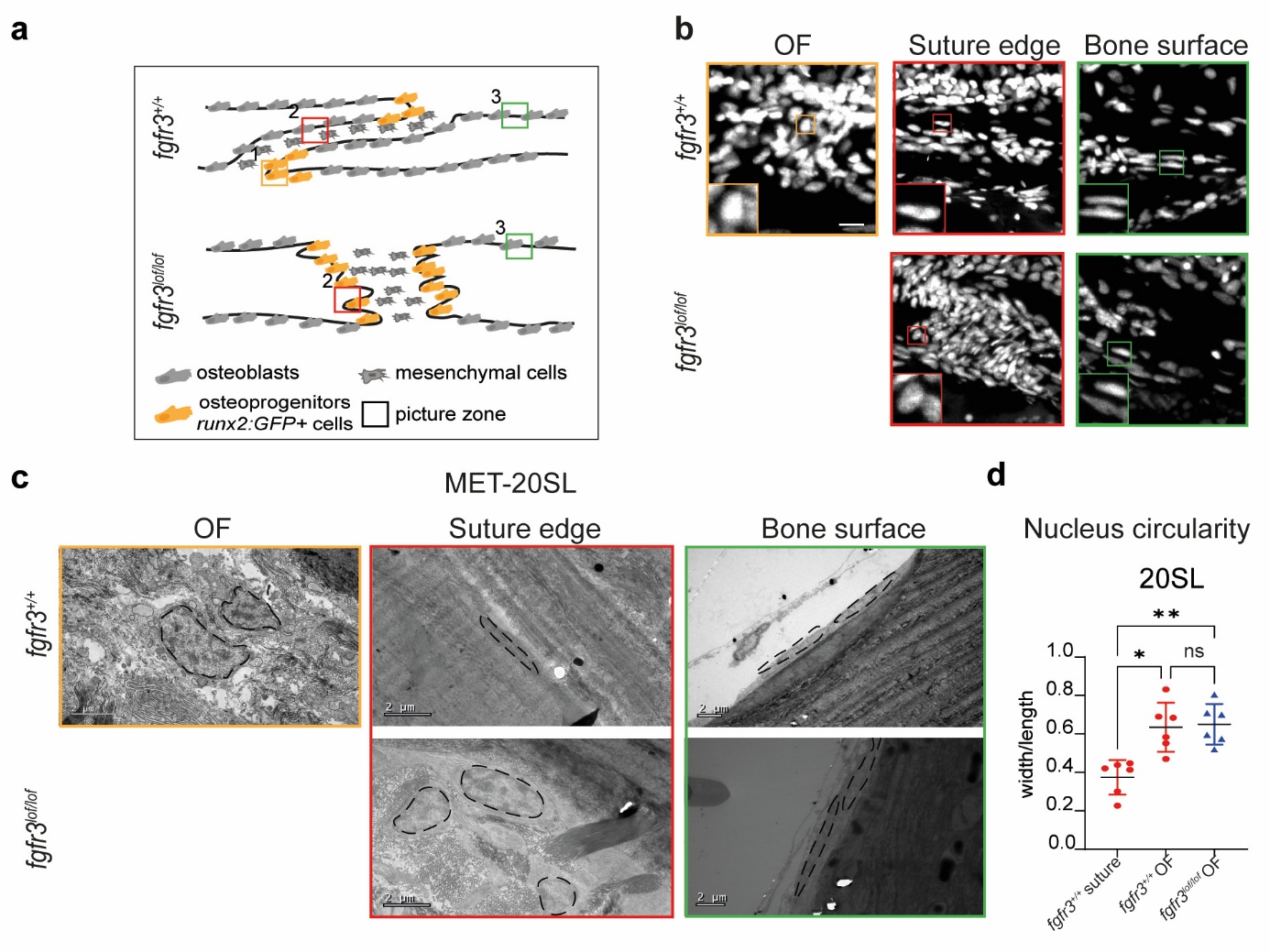


**Figure S3. Fgfr3 is an inhibitor of osteoprogenitors expansion within the suture.** The nuclei of cells along the suture in *fgfr3^lof/lof^* fish exhibit the shape of osteoprogenitor nuclei. (a) Schematic representation of *fgfr3^+/+^* and *fgfr3^lof/lof^* metopic sutures indicating the different zones studied. (b) High magnification of nuclei stained with DAPI at the osteogenic front (OF) and along the metopic suture of 20 SL *Tg(runx2:GFP*; *fgfr3^+/+^ or ^lof/lof^* ) fish. Scale bar = 5 μm. (c) Coronal sections of 20 SL fish imaged by transmission electron microscopy. The dotted lines represent the nuclear boundaries. Scale bar = 2 μm. (d) Quantitative comparison between the nucleus of *fgfr3+/+* cells along the suture and the OF and of *fgfr3^lof/lof^* cells along the sutures (*fgfr3^+/+^* n=6; *fgfr3^lof/lof^* n=6). The nuclear shape of *fgfr3^lof/lof^* cells along the suture is similar to that of OF cells in the controls, and not to that of cells at the edge of the metopic suture in the controls. Data are presented as mean ± SD. The p-values were determined by Student’s t tests. ns: not significant; p < 0.05 = *; p < 0.01 = **.


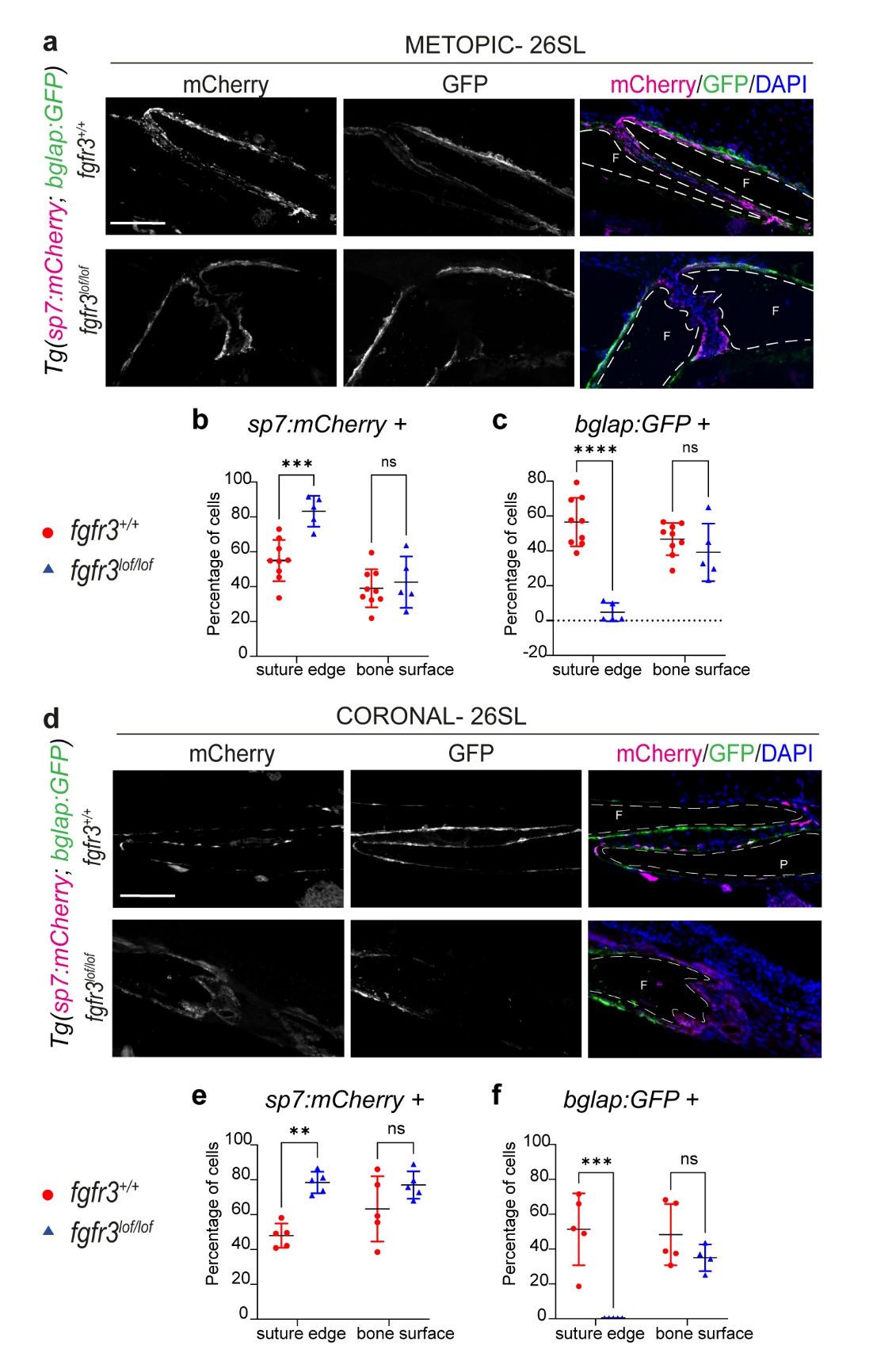


**Figure S4. Fgfr3 loss alters osteoblast maturation across coronal and metopic sutures in adulthood** (a) Immunofluorescence against GFP (green) and mCherry (red) was performed on metopic sutures of *Tg(sp7:mCherry; bglap:GFP*;

*fgfr3^+/+^ or ^lof/lof^*) fish at 26 SL. Nuclei were counterstained with DAPI (blue) and the dotted lines represent the bone boundaries. Scale bar = 50 μm. (b-c) Quantification at 26 SL of the percentage of *sp7*:mCherry positive cells and *bglap*:GFP positive cells along the suture and along the bone surface (*fgfr3^+/+^* n=9; *fgfr3^lof/lof^* n=5). (d) Immunofluorescence against GFP (green) and mCherry (red) was performed on coronal sutures of *Tg(sp7:mCherry; bglap:GFP*; *fgfr3^+/+^ or ^lof/lof^*) fish at 26 SL. Nuclei were counterstained with DAPI (blue) and the dotted lines represent the bone boundaries. Scale bar = 50 μm. (e-f) Quantification at 26 SL of the percentage of *sp7*:mCherry and *bglap*:GFP positive cells along the coronal suture (*fgfr3^+/+^* n=5; *fgfr3^lof/lof^* n=5; except *bglap* along the bone surface *fgfr3^lof/lof^* n=4). F: Frontal bone. P: Parietal bone. The p-values were determined by two-way ANOVA. ns: not significant; p < 0.01 = **; p < 0.001 = ***; p < 0.0001 = ****.


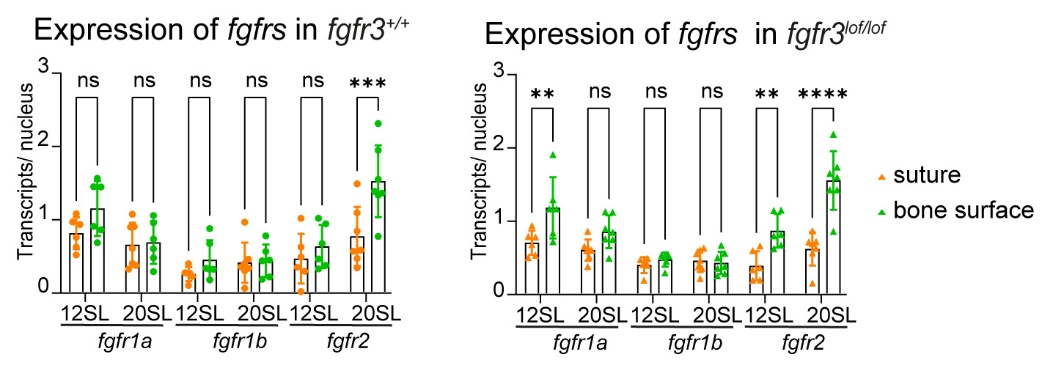


**Figure S5. *fgfr2* shows elevated expression at the bone surface.** RNAscope quantitative analyses of *fgfr1a, fgfr1b* and *fgfr2* expression in suture versus bone surface in *fgfr3^+/+^* or *fgfr3^lof/lof^* at 12 and 20 SL showed that *fgfr1a is* more highly expressed along the bone surface than within the suture in *fgfr3^lof/lof^* fish at 12 SL, while *fgfr2* is more highly expressed in bone surface than the suture at 20 SL, in both genotypes (*fgfr1a* and *fgfr1b*: 12 SL: *fgfr3^+/+^* n=6; *fgfr3^lof/lof^* n=6; 20 SL: *fgfr3^+/+^* n=7; *fgfr3^lof/lof^* n=6) (*fgfr2*: 12 SL: *fgfr3^+/+^* n=6; *fgfr3^lof/lof^* n=6; 20 SL: *fgfr3^+/+^* n=7; *fgfr3l^of/lof^* n=7). Data are presented as mean ± SD. The p-values were determined by two-way ANOVA. ns: not significant; p < 0.01 = **; p < 0.001 = ***; p < 0.0001 = ****. The dotted lines represent the bone boundaries.


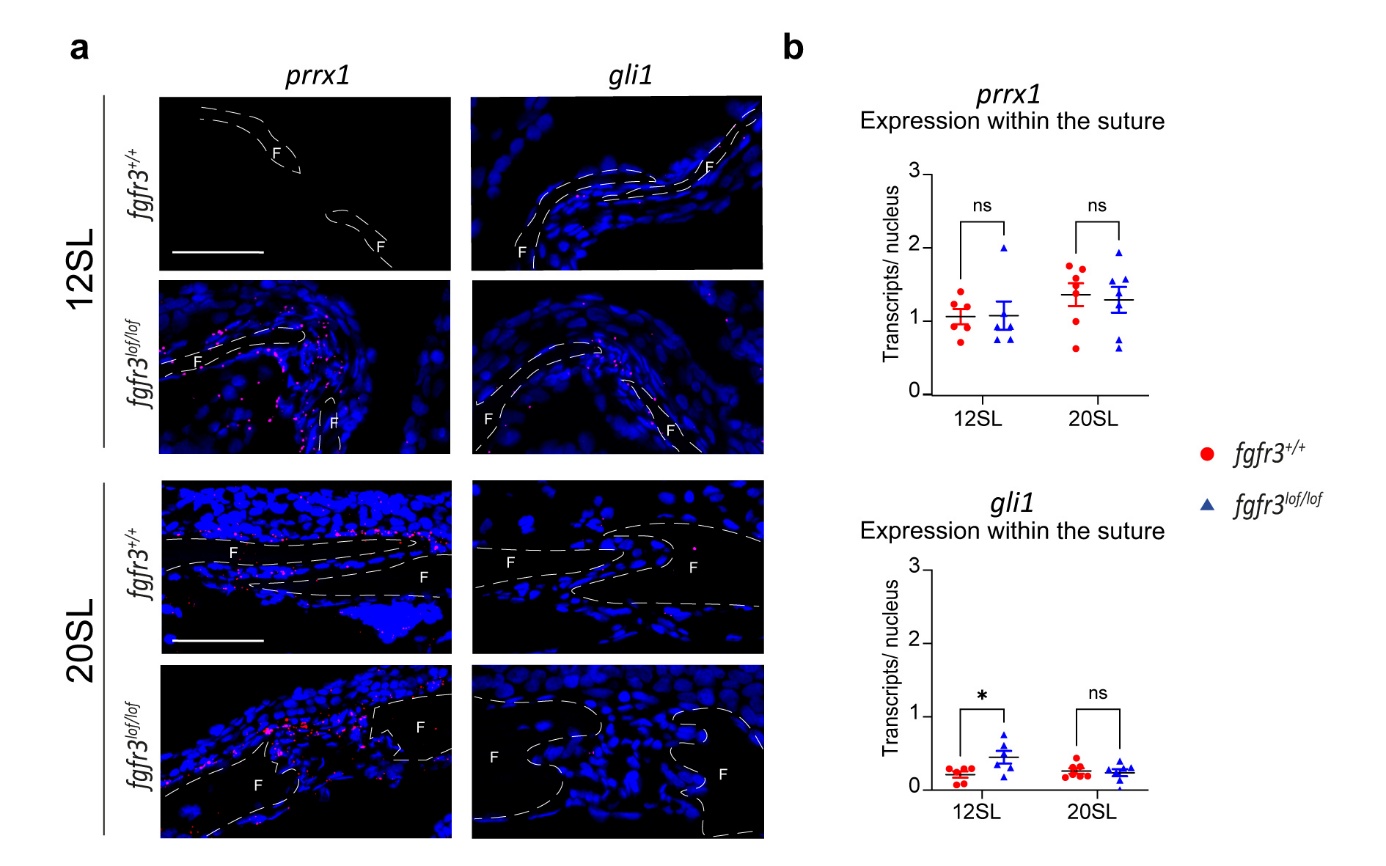


**Figure S6. *gli1 and prrx1 expression during cranial suture formation*** (a) Expression of *prrx1* and *gli1* (red) in coronal sections of the metopic suture at 20 SL, labeled by RNAscope *in situ* hybridization. Nuclei are counterstained with DAPI (blue). Scale bar = 50 μm. (b) Quantitative RNAscope analysis revealed that *prrx1* is not differentially expressed in *fgfr3^lof/lof^* compared to controls and a slight increased expression of *gli1* in *fgfr3^lof/lof^* metopic sutures compared to controls at 12 SL (12 SL: *fgfr3^+/+^* n=6; *fgfr3^lof/lof^* n=6; 20 SL: *fgfr3^+/+^* n=7; *fgfr3^lof/lof^* n=7). Data are presented as mean ± SD. F: Frontal bone. p-values were determined by two-way ANOVA. ns: not significant; p < 0.05 = *. The dotted lines represent the bone boundaries.
